# Uptake mechanism of iron-phytosiderophore from the soil based on the structure of yellow stripe transporter

**DOI:** 10.1038/s41467-022-34930-1

**Published:** 2022-11-23

**Authors:** Atsushi Yamagata, Yoshiko Murata, Kosuke Namba, Tohru Terada, Shuya Fukai, Mikako Shirouzu

**Affiliations:** 1 Laboratory for Protein Functional and Structural Biology, RIKEN Center for Biosystems Dynamics Research, 1-7-22 Suehiro-cho, Tsurumi-ku, Yokohama, Kanagawa Japan; 2 Bioorganic Research Institute, Suntory Foundation for Life Sciences, 8-1-1 Seikadai, Seika-cho, Soraku-gun, Kyoto, Japan; 3 Department of Pharmaceutical Sciences, Tokushima University, 1-78-1 Shoumachi, Tokushima-shi, Tokushima, Japan; 4 Department of Biotechnology, Graduate School of Agricultural and Life Sciences, The University of Tokyo, 1-1-1 Yayoi, Bunkyo-ku, Tokyo Japan; 5 Department of Chemistry, Graduate School of Science, Kyoto University, Kitashirakawa-Oiwakecho, Sakyo-ku, Kyoto, Japan

**Keywords:** Cryoelectron microscopy, Iron, Plant physiology, Membrane proteins, Permeation and transport

## Abstract

Calcareous soils cover one-third of all land and cause severe growth defects in plants due to the poor water solubility of iron at high pH. Poaceae species use a unique chelation strategy, whereby plants secrete a high-affinity metal chelator, known as phytosiderophores (mugineic acids), and reabsorb the iron-phytosiderophore complex by the yellow stripe 1/yellow stripe 1-like (YS1/YSL) transporter for efficient uptake of iron from the soil. Here, we present three cryo-electron microscopy structures of barley YS1 (HvYS1) in the apo state, in complex with an iron-phytosiderophore complex, Fe(III)-deoxymugineic acid (Fe(III)–DMA), and in complex with the iron-bound synthetic DMA analog (Fe(III)–PDMA). The structures reveal a homodimeric assembly mediated through an anti-parallel β-sheet interaction with cholesterol hemisuccinate. Each protomer adopts an outward open conformation, and Fe(III)–DMA is bound near the extracellular space in the central cavity. Fe(III)–PDMA occupies the same binding site as Fe(III)–DMA, demonstrating that PDMA can function as a potent fertilizer in an essentially identical manner to DMA. Our results provide a structural framework for iron-phytosiderophore recognition and transport by YS1/YSL transporters, which will enable the rational design of new, high-potency fertilizers.

## Abstract (web summary)

Iron is an essential mineral in plant physiology. YS1 transporter imports iron–phytosiderophore complex (Fe(III)–DMA) from the soil. Here, the authors describe the cryo-EM structures of barley YS1 in complex with substrate, and a synthetic substrate analog.

## Introduction

**Fig. 1:**
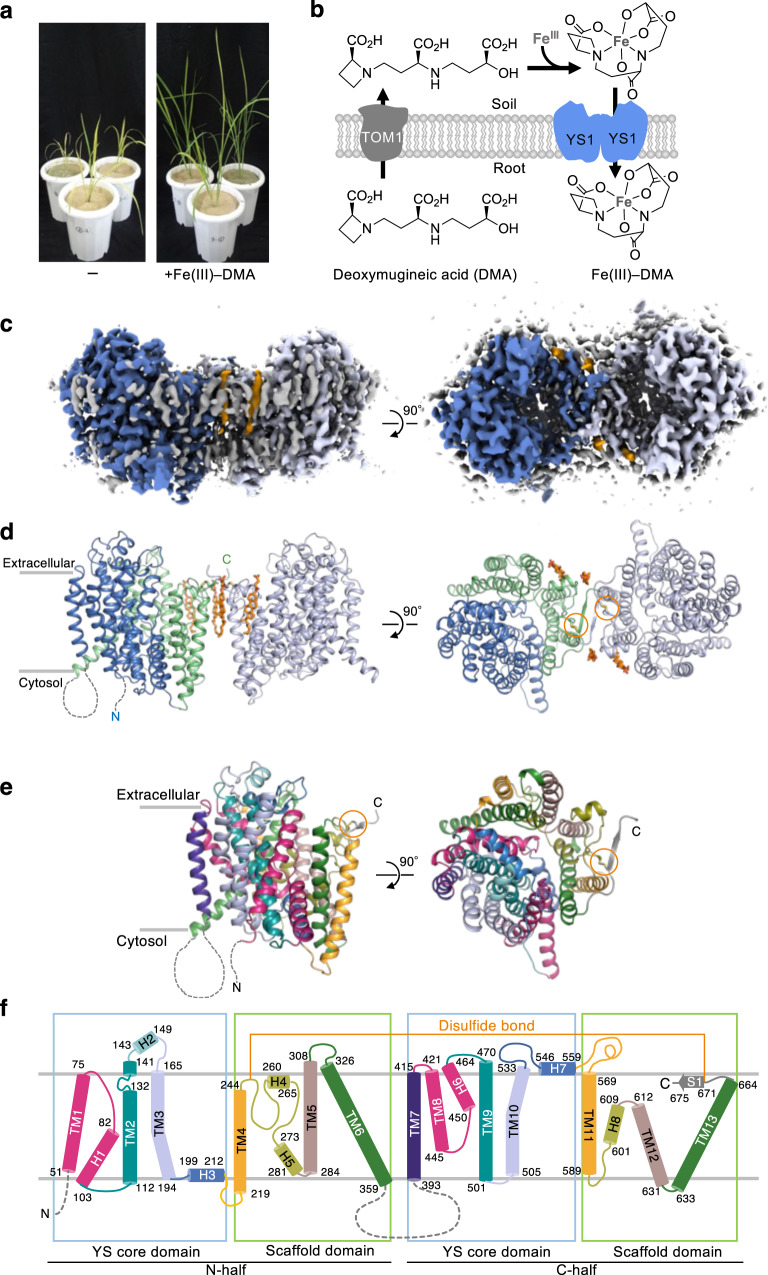
Cryo-EM structure of HvYS1 dimer. **a** Improvement of rice growth by DMA in alkaline soil. The pictures show rice grown in alkaline soil in the absence (left) or presence (right) of chemically synthesized DMA. **b** Schematic drawing of the plant phytosiderophore-dependent iron uptake mechanism. DMA is exported by TOM1 and Fe(III)–DMA is imported by YS1/YSL. **c** Cryo-EM density of the HvYS1 dimer at 2.9 Å resolution. Each protomer is colored in blue and light purple, respectively. The bound CHS molecules are highlighted in orange. **d** Side (left) and top (right) views of the structure of the HvYS1 dimer. The YS core domain is colored in blue and the scaffold domain in green. The CHS molecules (orange) and the disulfide bonds are shown in sticks. **e** Side (left) and top (right) views of the structure of the HvYS1 monomer structure. The coloring scheme is the same as that in **f**. The disulfide bond is shown in sticks and highlighted by the orange circle. **f** Topology diagram of HvYS1. The beginning and end of each secondary-structure element are numbered. The YS core and the scaffold domains are shown in the blue and green boxes, respectively.

Iron is an essential element for all living organisms, playing important roles as a cofactor for the catalysis of oxidation/reduction reactions in a wide variety of biological processes that include respiration and photosynthesis^1^. However, ferrous (Fe^2+^) ions are oxidized to sparingly soluble ferric (Fe^3+^) ions in high-pH calcareous soils. Therefore, most plants cannot efficiently absorb iron from calcareous soils, resulting in iron deficiency-induced chlorosis and severe plant growth defects^2^. Calcareous soils are widespread in dryland and cover more than 30% of the global land. Thus, the cultivation of calcareous soils is increasingly important and challenging. Poaceae species, including some of the major crops, e.g., maize, rice, wheat, and barely, secret mugineic acids family phytosiderophores (MAs) for efficient uptake of ferric ions from calcareous soils, whereby they are generally less sensitive to iron deficiency^3–5^. MAs form highly water-soluble complexes with the various metal ions at an equimolar molar ratio. Intriguingly, rice is highly sensitive to iron deficiency because of its poor secretion of deoxymugineic acid (DMA). Nicotinamine (NA) is a biosynthetic precursor of MAs. Transgenic rice harboring barley NA aminotransferase genes, which are essential for the biosynthesis of MAs from NA, show increased secretion of MAs, which in turn leads to enhanced tolerance to low iron availability in alkaline soil^6^. Moreover, the application of synthetic DMA to the soil increases iron deficiency tolerance in rice without any genetic alteration (Fig. 1a)^7^. Chemically synthesized DMA derivatives with higher stability and lower cost have been developed and reportedly proved to function as well as DMA^7^. Nonetheless, developing more efficient DMA derivatives requires elucidation of the molecular mechanism underlying iron–phytosiderophores uptake from the soil.

MAs are biosynthesized from methionine in the roots^8,9^ and are secreted through the TOM1 transporter into the soil^10^. The yellow stripe 1/yellow stripe 1-like (YS1/YSL) family transporter reabsorbs the iron–MAs complex at the root surface^11,12^ (Fig. 1b). The name “yellow strip” is derived from the mutant phenotype showing leaf interveinal chlorosis in maize. Transport activity of Fe(III)–DMA by YS1/YSL was verified by a complementary assay using the heterologous expression of YS1 in the yeast mutant, which is defective in its endogenous iron uptake pathways, or in *Xenopus* oocytes^11,12^. Barley YS1 (*Hordeum vulgare L*. YS1, HvYS1) predominantly transport Fe(III)–MAs complex, whereas Maize YS1 (*Zea mays L*. YS1, ZmYS1) shows broader substrate specificity for various metal ions, such as Fe(III), Fe(II), Ni(II), Co(II), Cu(II), and Zn(II)^12^. In addition, the yeast complementary assay demonstrated that YS1 is a proton-coupled symporter for Fe(III)–DMA^13^. Following the identification of YS1 transporter from maize in 2001^11^, hundreds of YS1/YSL proteins, including the multiple paralogs, have been identified, indicating that the YS/YSL family forms a large family of plant transporters^14,15^. Nevertheless, the detailed mechanism of iron-phytosiderophore uptake remains unknown due to the lack of structural and biochemical analyses. In this work, we present the cryo-EM structures of HvYS1 in the apo state, in complex with Fe(III)–DMA, and in complex with the iron-bound synthetic DMA analog (Fe(III)–PDMA). Structure-based mutational analysis and computational simulations elucidated a proton-coupled elevator-like transporter mechanism by YS1 transporters.

## Results

### Cryo-electron microscopy structure of HvYS1

We focused on HvYS1, which reportedly shows strict specificity for Fe(III)–MAs^12^. HvYS1 was overexpressed in insect (*Spodoptera frugiperda* Sf9) cells and purified in the detergent lauryl maltose neopentyl glycol (LMNG) with cholesterol hemisuccinate (CHS). The purified HvYS1 predominantly exists as a dimer, whereas it exists as a monomer when purified in the absence of CHS (Supplementary Fig. 1a). CHS further enhances the thermal stability of HvYS1 (Supplementary Fig. 1b). These effects seemed to be specific to CHS, as the soy extract polar lipid did not show any effect on both the dimerization and stabilization of HvYS1 (Supplementary Fig. 1a, b). The dimeric HvYS1 was analyzed by single particle analysis with cryo-electron microscopy. The cryo-EM map was reconstructed to an overall resolution of 2.9 Å with an imposed *C*2 symmetry (Fig. 1c, Supplementary Fig. 2, and Supplementary Table 1). The quality of the cryo-EM map was sufficient for the de novo building of a nearly complete atomic model (Supplementary Figs. 2, 3).

The structure of HvYS1 reveals a homodimeric structure (Fig. 1d). Additional densities, assigned to be CHS, are observed. Four CHS molecules within the HvYS1 dimer are assigned, and two of them are present between the protomers (see also “Dimer interface”; Fig. 1d and Supplementary Fig. 1c). The cytoplasmic part of the two subunits interface is occupied by the lipid-like densities (Supplementary Fig. 1d). The first 47 residues and the linker between TM6 and TM7 were invisible owing to their flexibilities. Structurally, the HvYS1 protomer is subdivided into two domains (Fig. 1d–f). One is the core domain called the “YS core” domain, while the other is a scaffold domain that cradles the YS core and comprises the dimer interface. Both domains showed no significant structural homologs in a DALI server search^16^. The YS core domain is composed of N- and C-halves; TM1–H1, TM2, TM3, and H3 in N-half are topologically related to TM8–H6, TM9, TM10, and H7 in C-half by an inverted repeat symmetry (Supplementary Fig. 4a). TM1–H1 and TM8–H6 form a helical hairpin in which two helices are separated by an extended loop, unlike the typical helical hairpins in which two closely spaced helices are connected by a short turn (Supplementary Fig. 4b). TM2 forms the discontinuous helix. The helical hairpin and the discontinuous helix are likely to contribute to the substrate-binding in analogy with the other membrane transporters (Supplementary Fig. 4b)^17^. The N- and C-halves of the YS core are connected to those of the scaffold domain via the near-horizontal ‘arm’ helices, H3 and H7, respectively. The scaffold domain is also composed of symmetry-related inverted repeats (Supplementary Fig. 4c). The N-terminal half of the scaffold domain forms an “M” shape, whereas the C-terminal half forms a “W” shape (Supplementary Fig. 4c). The “M”- and “W”-shaped repeats cross each other to form extensive contact between them (Supplementary Fig. 4c). The C-terminal end of the scaffold domain folds to a strand (S1) that is oriented parallel to the membrane (Fig. 1d.; Supplementary Fig. 4c). The N- and C-halves in the scaffold domain are pinned through a disulfide bond between Cys250 and Cys671 (Fig. 1e, f; Supplementary Fig. 4c). The YS core and the scaffold domains together constitute a large central cavity. The overall structure of the HvYS1 protomer represents an outward open conformation, as the central cavity is wide open to the extracellular space, whereas the base of the cavity is sealed from the cytoplasm.

### Dimer interface

**Fig. 2:**
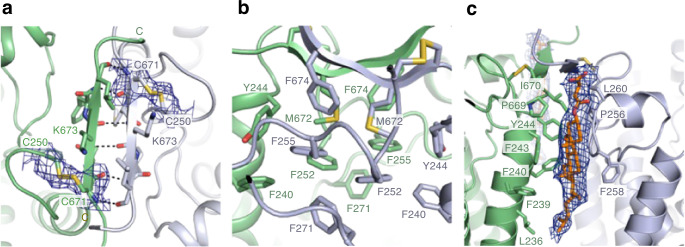
Dimer interface. **a** The dimer interface is mediated through an anti-parallel β-sheet. The hydrogen bonds formed by the anti-parallel β-sheet are shown as dashed lines. The cryo-EM density of the disulfide bond formed between Cys250 and Cys671 is shown as a blue mesh. **b** Van der Waals contacts in the dimer interface. The residues contributing to van der Waals contacts are indicated by sticks. **c** CHS molecules are bound between HvYS1 dimers. The cryo-EM densities of CHS molecules at the dimer interface are shown as a blue mesh. The residues interacting with CHS are indicated by sticks.

The scaffold domains from each protomer comprise the dimer interface with a buried surface area of 936 Å^2^. Each protomer’s C-terminal β -strands (S1s) form an anti-parallel β-sheet to assemble the dimer (Fig. 2a). In addition, a cluster of the hydrophobic residues including Phe240 and Tyr244 on TM4, Phe252 and Phe255 in the TM4–H4 loop, Phe271 in the H4–H5 loop, Met672 and Phe674 on S1, pack together to stabilize the dimer (Fig. 2b). The disulfide bond (Cys250–Cys671), connecting S1 and the loop between H4–TM4, is likely to enhance the rigidity of the dimer interface structure (Fig. 2a). The cysteine pairs (Cys250–Cys671) and the hydrophobic residues comprising the dimer interface are highly conserved within the YS/YSL proteins (Supplementary Fig. 5), suggesting a shared homodimeric assembly in this group of proteins. Two interfacial CHSs are surrounded by the highly conserved hydrophobic residues (Fig. 2c and Supplementary Fig. 5).

To verify the dimer interface observed in the cryo-EM structure, we introduced a cysteine mutation at Lys673 in the middle of the S1 (Fig. 2a). The purified HvYS1^K673C^ spontaneously formed the disulfide crosslinked dimer in the non-reducing polyacrylamide gel electrophoresis (Supplementary Fig. 6a–c). The crosslinked dimer was enhanced by oxidant, CuCl_2_, and was almost completely reversed by reducing reagent, DTT. When HvYS1^K673C^ was purified without CHS, both dimer and monomer were detected (Supplementary Fig. 6a). The dimer of HvYS1 was spontaneously formed presumably with the endogenous cholesterol in the insect cell membrane. The crosslinked dimer of HvYS1^K673C^ was retained nevertheless the delipidation by detergents.

### Fe(III)–DMA-binding site

**Fig. 3:**
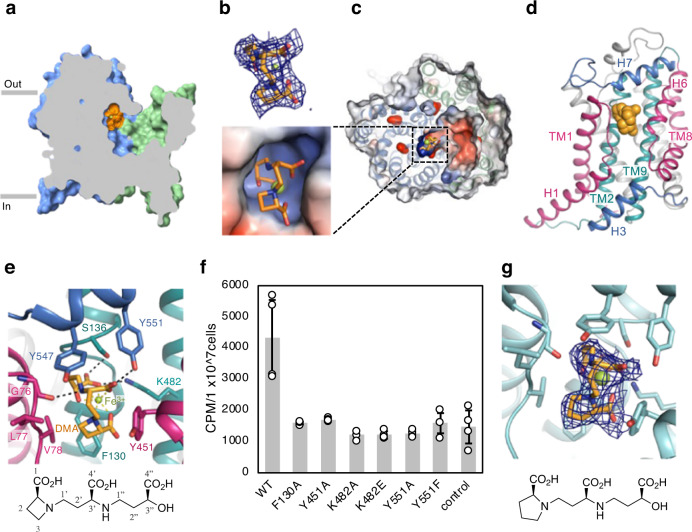
Fe(III)–DMA binding site. **a** Cutaway surface representation of the HvYS1 protomer structure in complex with Fe(III)–DMA. HvYS1 is in outward-open conformation. Fe(III)–DMA (orange) is bound near the extracellular space. **b** The cryo-EM density (blue mesh) of the bound Fe(III)–DMA at a contour level of 5σ. **c** Electrostatic surface representation of the central cavity and the substrate binding pocket. DMA is indicated by sticks (DMA) and Fe^3+^ is shown by the green sphere. **d** Fe(III)–DMA binding site viewed from the scaffold domain. The inverted repeats of the helix hairpin and the subsequent central TM are colored in warm pink and deep teal, respectively. **e** Close-up view of the Fe(III)–DMA binding site. The residues interacting with Fe(III)–DMA are indicated by sticks. **f** Uptake of ^55^Fe upon adding ^55^Fe-DMA to the insect cells that expressed wild-type (WT) or a mutant of HvYS1. Control is uninfected insect cells. CPM stands for counts per minute. Data are shown as means ± standard deviations (SD); *n*  =   4 biologically independent experiments. Data between WT and mutants were analyzed by two-sided test (F130A (*p* = 0.0300), Y451A (*p* = 0.0362), K482A (*p* = 0.0270), K482E (*p* = 0.0198), Y551A (*p* = 0.0184) and Y551F (*p* = 0.0301)). Source data are provided as a Source Data file. **g** Fe(III)–PDMA binding site structure. Cryo-EM density (blue mesh) of bound Fe(III)–PDMA, contoured at 5σ, is shown.

To elucidate the substrate recognition and transport mechanism, we determined the cryo-EM structure of HvYS1 in complex with Fe(III)–DMA to an overall resolution of 2.7 Å (Supplementary Figs. 7, 8 and Supplementary Table 1). Our efficient synthesis method enabled us to prepare a sufficient amount of DMA for the cryo-EM analysis^18^. The overall structure of HvYS1 in complex with Fe(III)–DMA is essentially identical to that in the apo state, with rmsd of 0.4 Å for Cα atoms. The unambiguously assigned Fe(III)–DMA density is found near the extracellular space in the YS core domain (Fig. 3a, b). The bound Fe(III)–DMA, in which Fe(III) is octahedrally coordinated, adopts a packed conformation similar to those of the inorganic crystal structures of Cu(II)–MA and Co(II)–MA (Supplementary Fig. 9a)^19^. As the cavity is negatively charged, the Fe(III)–DMA containing three carboxyl groups is exclusively bound to the positively charged pocket (Fig. 3c). The binding pocket consists of the TM1–H1 loop, TM2, TM9, H6, and juxtamembrane H7 (Fig. 3d), extending into the unwound part of TM2 formed by the conserved Gly132, Gly133, and Gly135 (Supplementary Fig. 9b). The backbone of Fe(III)–DMA is predominantly surrounded by the hydrophobic residues, Leu77, Val78, Phe130, Tyr451, Tyr547, and Tyr551 (Fig. 3e). Particularly, Tyr547 in the juxtamembrane H7 appears to cap the binding pocket. In addition, hydrogen bonds support the specific interaction with the packed backbone structure of the Fe(III)–DMA complex. The 4”-carboxyl group of DMA forms a salt bridge and hydrogen bond with Lys482 on TM9 and Ser136 on TM2, respectively. The secondary amino group and the 4’-carboxyl group of DMA form hydrogen bonds with the carbonyl group of Gly76 and the side chain of Tyr551, respectively. Further, the four-membered ring of DMA forms van der Waals contacts with Val78, Phe130, and Tyr451. The side chain of Tyr451 in the apo structure points inside to occupy the Fe(III)–DMA binding site (Supplementary Fig. 9c). No coordination of Fe(III) by the amino acid residues in HvYS1 is observed, consistently with the broad specificity of YS1/YSL proteins for the various metal ions.

To validate the importance of the binding interface residues, we analyzed the radioactive ^55^Fe–DMA uptake by the insect cells heterologously expressing either the wild-type or the mutant HvYS1s^7^ (Fig. 3f). The insect cells expressing the wild-type HvYS1 effectively transported Fe(III)–DMA from outside into the cells. In contrast, and consistently with the elucidated cryo-EM structure, all mutant HvYS1 (F130A, Y451A, K482A, K482E, Y551A, and Y551F) reduced their transport activities to a background level. The residues tested by mutagenesis are strictly conserved within four YS1/YSL proteins (HvYS1, ZmYS1, rice YSL15, and barley YSL2), which were shown to transport metal–DMA complex^20–22^ (Supplementary Fig. 5). However, either Phe130 or Tyr551 is not conserved in the other three YSL proteins (rice YSL2, *Arabidopsis thaliana* YSL1, and *Thlaspi caerulescens* YSL3), which transport metal–NA complex^23–25^ (Supplementary Fig. 5). RiceYSL2 is unable to transport Fe(III)–DMA^23^. MAs are produced in Poaceae, not in Brassicaceae containing *A. thaliana* and *T. caerulescens*. Therefore, these three YSL proteins should be NA-specific transporters and might recognize the metal–NA complex in a slightly different way from that in the HvYS1 structure.

### The synthetic phytosiderophore analog

The extent of plant growth improvement achieved by spraying synthetic DMA to alkaline/calcareous soils promises a successful agricultural application. However, the four-membered ring of DMA has a highly strained structure, causing poor stability of MAs. In addition, it is supplied from the expensive l-azetidine-2-carboxylic acid. To overcome these limitations of DMA, proline 2’-deoxymugineic acid (PDMA), a DMA analog, was synthesized by replacing the four-membered azetidine ring with a five-membered ring^7^. PDMA effectively substituted DMA for iron uptake by HvYS1 in the insect-cell assay system, and significantly improved the growth of rice in calcareous soil^7^.

In order to verify if PDMA functions in YS1 in the same manner as DMA, we determined the cryo-EM structure of HvYS1 in complex with Fe(III)–PDMA to an overall resolution of 2.9 Å (Supplementary Figs. 10, 11). The HvYS1 structure bound to Fe(III)–PDMA reveals the outward open conformation, nearly identical to the apo and the Fe(III)–DMA bound structures. Fe(III)–PDMA occupies the Fe(III)–DMA-binding pocket (Fig. 3g). The carboxyl and amino group moieties of PDMA are recognized by YS1 in the same manner as those of DMA. The five-membered ring of PDMA is well fit with the binding pocket for the four-membered ring without any steric clashes. Our cryo-EM data supports that PDMA activity as a potent fertilizer uses the same mechanism as DMA.

### Elevator-like transport mechanism

**Fig. 4:**
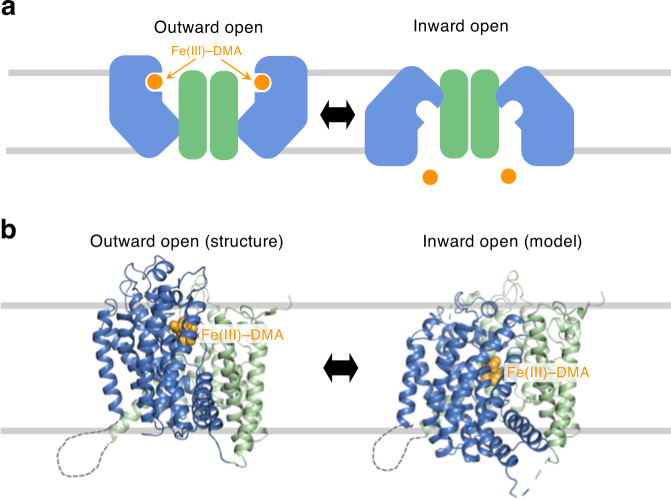
Conformational transition from outward open to inward open. **a** The schematic drawing of the elevator-like transport mechanism of HvYS1. The YS core and the scaffold domains are colored in blue and green, respectively. Fe(III)–DMA is shown in orange spheres. **b** Outward open structure (left) and inward open model (right). For clarity, only one subunit is shown.

HvYS1 comprises two distinct domains: a substrate-binding YS core and a scaffold domain, implicating an elevator-like transport^17^. Either dimeric or trimeric transporters, which consist of a transporting (core) domain and a scaffold domain mediating oligomerization, share an elevator-like mechanism (Fig. 4a). In an elevator-like transport, a rigid body movement of the substrate-binding core domain against the anchored scaffold domain allows the translocation of the bound substrate across the membrane, typically 10–18 Å^26–28^. Fe(III)–DMA is bound at ~10 Å upward from the center of the lipid bilayer. Similar to modeling an inward open conformation of the bacterial glutamate transporter^29^, an inward open model of HvYS1 was generated by swapping the inverted repeats of the N- and the C-halves in each the YS core or the scaffold domains (Fig. 4b). Fe(III)–DMA is shifted ~15 Å towards the cytoplasm than the outward open conformation.

### Proton-coupled mechanism

**Fig. 5:**
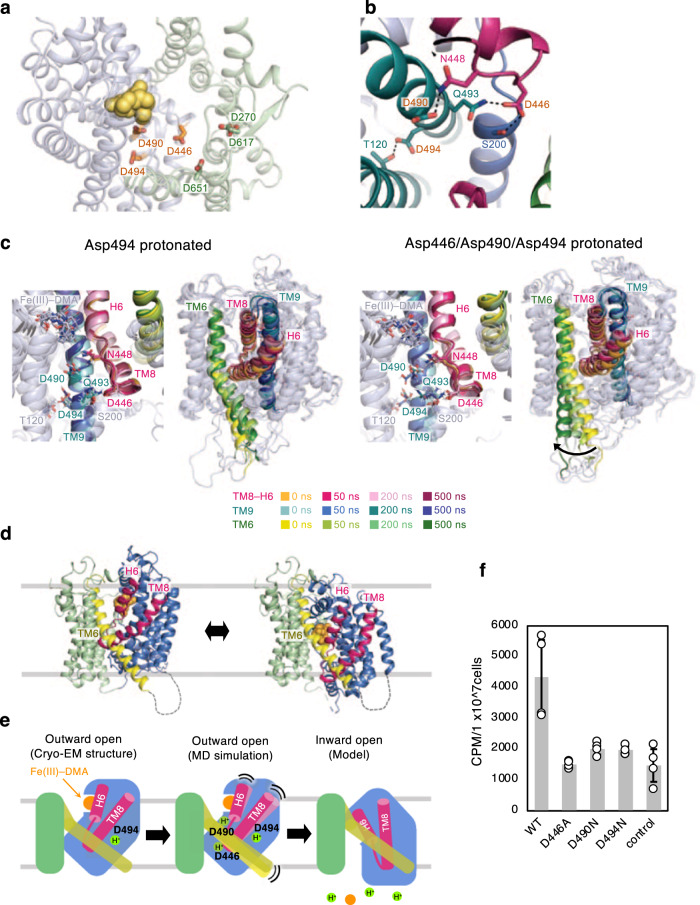
Potential proton-binding sites for transport. **a** The Asp residues in the cavity. **b** The interhelical H-bonds by three Asp residues. **c** MD simulations with Asp494 protonated (left) and those with Asp446/Asp490/Asp494 protonated. The interhelix H-bonds with three Asp residues and their conformations in MD simulations are shown in the left. The fluctuation of TM6, TM8–H6 in MD simulations are shown in the right. **d** Outward open structure (left) and inward open model (right) with 140° rotated from Fig. 4b. TM6 and TM8–H6 are colored in yellow and hotpink, respectively. **e** The schematic drawing of the proton-driven transport mechanism of HvYS1. **f** The insect cell-based transport assay of the wild-type and the mutant HvYS1s. The experimental procedures are as same as Fig. 3f. Data are shown as means ± standard deviations (SD); *n* = 4 biologically independent experiments. Data between WT and mutants were analyzed by two-sided test (F446A (*p* = 0.0300), D490N (*p* = 0.0369), and D494N (*p* = 0.0374)). Source data are provided as a Source Data file.

HvYS1 is a proton-driven symporter^13^. To gain insight into the proton binding site inside the cavity for proton-coupled transport, we performed MD simulations. Within all titratable residues in HvYS1, six Asp residues (Asp270, Asp446, Asp490, Asp494, Asp617, Asp651) are on the surface of the central cavity of HvYS1 (Fig. 5a; Supplementary Fig. 12a). The ion- or proton-binding sites and substrate-binding site are within the substrate-binding core domain in elevator transporters (Fig. 1f; Supplementary Fig. 4b)^17^. Therefore, we focused on the protonation of Asp446, Asp490, and Asp494, which are located inside the YS core domain. In the cryo-EM structure, these three Asp residues form an H-bond network to stabilize the configuration of the TM8–H6 loop and TM9 (Fig. 5b). We first tested each protonation of these three Asp residues to identify the protonation state of the cryo-EM structure. In the MD simulations, HvYS1 was embedded in the POPC membrane, and four CHS molecules were replaced with cholesterol. The distances between the Fe(III) ion and the ligand atoms were harmonically restrained to maintain the Fe(III)–DMA conformation. As a result, the simulation with Asp494 protonated is remarkably stable, as the rmsd of the bound Fe(III)–DMA shows minimum fluctuation (Supplementary Fig. 12c), suggesting that the Asp494 protonated state structure corresponds to the cryo-EM structure.

The protonation would function not only to stabilize the substrate-bound state but also to induce the conformational change necessary for transport. We next examined the effect of additional protonation to that on the Asp494 protonated state to identify the protonation sites triggering the conformational change. Among three additional protonated states (Asp446/Asp494 protonated, Asp490/Asp494 pronated, and Asp446/Asp490/Asp494 protonated states), only the simulation with Asp446/Asp490/Asp494 protonated displays a substantial conformational change as revealed by the principal component analysis performed for the MD trajectories of the helical segments (Supplementary Figs. 12b; 13). The largest fluctuation occurs on the TM6 in the simulation with Asp446/Asp490/Asp494 protonated (Fig. 5c; Supplementary Fig. 12c). TM6 moves away from YS core domain, suggesting a weaker interaction between TM6 and YS core domain, which would enable the YS core to slide along TM6, leading to the structural transition from an outward open to an inward open conformation (Fig. 5d). Taken together, we propose an elevator-like mechanism of iron-phytosiderophore transport coupled with a proton-binding by YS1/YSL family proteins (Fig. 5e). To support the functional importance of Asp446, Asp490, and Asp494, we confirmed that each mutation of them impaired the transport activity in insect cell-based assay (Fig. 5f). Apparently, further structures in the different conformations are necessary to clarify how the proton is coupled to the global structural transition. The present high-resolution cryo-EM structures, together with the computational analyses, provide mechanistic insight into the elevator-like transport mechanism by YS transporters.

## Discussion

Our structural and functional studies revealed a homodimeric assembly of HvYS1 stabilized by CHS. A wide variety of phytosterols, such as campesterol, stigmasterol, and sitosterol, are the major sterols in plants. Intriguingly, phytosterol is highly enriched in the plasma membrane of the barley roots^30,31^. HvYS1 binds to CHS through hydrophobic interactions with the sterol. Phytosterol also should bind to HvYS1 as same as CHS and support the dimeric assembly. Gupta et al. proposed that interfacial lipids are required to stabilize the weak interface of the oligomeric membrane protein^32^. The buried surface area of 936 Å^2^ in the HvYS1 dimer interface is a considerably weak interface. Concomitantly, the interfacial CHS is required to preserve the dimeric interface of HvYS1. HvYS1 is likely to be functional as a dimer, because CHS remarkably enhances the thermal stability of HvYS1. However, HvYS1 should be in the equilibrium between dimer and monomer in the membrane in our insect cell-based assay system. In an elevator-like transport model, the transport activity is not necessary to be coupled with the oligomerization, though the oligomerization might contribute to the structural rigidity as a scaffold. Taken together, we could not rule out the possibility of HvYS1 working as a monomer in our experiment.

HvYS1 creates the deep binding pocket to accommodate Fe(III)–DMA. The binding site structure is highly specific to the configuration of the DMA backbone bound to Fe(III). No structural information on free DMA has been available so far. Free DMA is predicted to form an elongated conformation, precluding specific recognition by HvYS1. Our computational simulations identify Asp446, Asp490, and Asp494 as potential proton-binding sites. The protonation at Asp494 would be important to stabilize the substrate-bound state, whereas the additional protonation at Asp446 and Asp490 would trigger the conformational change from outward-open to inward-open form. Asp446 and Asp490 form the interhelical H-bonds with Gln493 and Asn448, respectively, to stabilize the configuration between TM8–H6 and TM9. In our simulation, the protonation at both Asp446 and Asp490 disrupts the interhelical H-bonds, presumably leading to the larger fluctuation of TM8 (Fig. 5c). As TM8 contacts with TM6, the larger fluctuation of TM8 would lead to the displacement of TM6 from YS core, triggering the conformational change from outward-open to inward-open form (Fig. 5c–e).

Given the continuous growth of the human population and the foreseeable food shortage, the cultivation of widespread calcareous soils is rapidly becoming a high priority to ensure global food security in the future. Since mugineic acid was discovered nearly half a century ago^33^, many efforts, including the chemical synthesis of MAs and their stable derivatives as well as the trans-genetic approaches, have been devoted to the practical application of MAs^6,7,18^. Our work provides a structural framework of YS transporter architecture and illustrates how mugineic acids family phytosiderophore function in iron uptake at the atomic level. In addition, we demonstrated that PDMA could be a potent fertilizer with the same functionality as DMA. These results will enable the structure-based design of phytosiderophore analogs to be used as potent fertilizers in agriculture.

## Methods

### Protein expression and purification

The gene encoding *Hordeum vulgare L*. YS1 (HvYS1) was PCR amplified and cloned into the pFastBac Dual vector. HvYS1 fused with the C-terminal octahistidine tag was transformed into *Escherichia coli* DH10multibac competent cells to generate the baculovirus DNA using a bac-to-bac system (Thermo Fisher). The baculovirus was generated in *Spodoptera frugiperda* Sf9 insect cells. Proteins were expressed in the Sf9 cells at 27 °C for 72 h. The cell pellets were dissolved in a lysis buffer containing 20 mM Tris-HCl (pH 8.0), 300 mM NaCl, 10% glycerol, and homogenized using a Dounce homogenizer. After ultracentrifugation at 186,000 × *g* at 4 °C for 40 min, the membrane pellet was solubilized in a lysis buffer containing 1% lauryl maltose neopentyl glycol (LMNG) and 0.2% cholesterol hemisuccinate (CHS) at 4 °C for 2 h. We added CHS in the purification step because cholesterol was well known to modulate the stability and function of membrane proteins including GPCRs and ion channels^34^. After centrifugation at 186,000 × *g* for 40 min, the supernatant was collected and applied to Ni-NTA (QIAGEN) resin. The resin was washed with 10 column volumes of buffer containing 50 mM imidazole, 20 mM Tris-HCl (pH 8.0), 300 mM NaCl, 10% glycerol, 0.001% LMNG, and 0.0002% CHS. The bound protein was eluted with buffer containing 500 mM imidazole, 20 mM Tris-HCl (pH 8.0), 300 mM NaCl, 10% glycerol, 0.001% LMNG, and 0.0002% CHS. Then, the protein was further purified by size-exclusion chromatography using a Superdex 200 10/300 GL (Cytiva) preequilibrated with the SEC buffer containing 20 mM HEPES (pH 7.5), 150 mM NaCl, 0.001% LMNG, and 0.0002% CHS.

HvYS1 without additional lipids was purified in the same manner as that with CHS, except that no supplemental lipid was added throughout the purification. HvYS1 with the soy extract polar lipid was purified in the same manner as that with CHS, except that 0.1 g/L of the soy extract polar lipid (avanti) was added instead of CHS. HvYS1s with/without CHS, with the soy extract polar lipids, were simultaneously purified from the same batch of Sf9 cultured cells.

### Fluorescence-based thermal stability assay

Thermal stability of HvYS1 was assessed by the modified CPM (N-[4-(7-diethylamino4-methyl-3-coumarinyl)phenyl]maleimide) assay^35^. CPM (Sigma) was diluted from the stock solution (4 g L^−1^ in DMSO) in a buffer used for the final size exclusion chromatography to be a concentration of 0.08 g L^−1^. A 20 µL of CPM solution was added to a 200 µL of HvYS1 in a 96-well plate, which was transferred to a microplate reader, SpectraMax ID3 (Molecular devices) prewarmed at 40 °C. The protein unfolding process occurring at 40 °C was monitored by CPM fluorescence at excitation and emission wavelength of 384 and 470 nm, respectively.

### Disulfide crosslinking

HvYS1^K673C^ with/without CHS were simultaneously purified from the same batch of Sf9 cultured cells, in the same manner as the wild-type protein. HvYS1^K673C^ with CHS was predominantly eluted as a dimer in SEC, whereas that without CHS was eluted as both dimer and monomer. The thermal stabilities of the dimeric HvYS1^K673C^ with CHS, the dimeric HvYS1^K673C^ without CHS, and the monomeric HvYS1^K673C^ without CHS were assessed with the fluorescence-based thermal stability assay described above.

The crosslinking experiments were carried out using HvYS1^K673C^ with CHS. In non-reducing SDS-PAGE with CBB-staining, the purified HvYS1^K673C^ spontaneously formed a crosslinked dimer, whereas the wild-type protein did not. An oxidant, CuCl_2_, was added to the purified HvYS1^K673C^ to a final concentration of 5 mM, and an enhancement of the crosslinked dimer was detected. The reducing reagent, DTT, was added to HvYS1^K673C^ to a final concentration of 50 mM, and the crosslinked dimer was almost completely reversed. In both, the wild-type protein was treated in the same manner as a negative control.

### Cryo-EM sample preparation and data collection

Purified HvYS1 was concentrated to ~4 mg mL^−1^ using a 100 kDa filter concentrator (Amicon). For HvYS1–Fe(III)–DMA and HvYS1–Fe(III)–PDMA complexes, ~10 µM of HvYS1 was mixed with either 100 µM of Fe(III)–DMA or 100 µM of Fe(III)–PDMA, respectively. After incubation on ice, the sample was concentrated to ~4 mg mL^−1^. A 3 µL of the sample was applied to a glow-discharged holey carbon grid (Quantifoil Cu 300 mesh, R1.2/1.3). The grid was blotted for 3 s and plunged into liquid ethane under 100% humidity at 4 °C using a Vitrobot Mark IV (Thermo Fisher).

Micrographs were acquired on a Titan Krios microscope (Thermo Fisher) operating at 300 kV equipped with a K3 direct electron detector (Gatan) and a Bioquantum energy filter with a slit width of 15 eV, at a magnification of 105,000×, resulting in a pixel size of 0.8285 Å, using EPU software (Thermo Fisher). A total of 5,278 movie frames for apo HvYS1 were captured using a total dose of 50.3 e^−^ Å^2^ for 50 frames with an exposure time of 2.35 s. In turn, a total of 11,930 movie frames for Fe(III)–DMA–HvYS1 complex were captured using a total dose of 51.4 e^−^ Å^2^ for 48 frames with an exposure time of 2.35 s. Lastly, a total of 6027 movie frames for the Fe(III)–PDMA–HvYS1 complex was captured using a total dose of 49.8 e^−^ Å^2^ for 48 frames with an exposure time of 2.20 s. Collected movie frames were imported into RELION 3.1^36,37^. The motion correction was performed using the MotionCorr2^38^, and the contrast transfer function (CTF) estimation was conducted with CTFFIND-4.1^39^.

Initial particle picking for apo HvYS1 was performed with crYOLO-1.7.6^40^, and the selected particles were used as a template for re-picking by TOPAZ^41^, resulting in a total of 2,276,022 particles. Particles were extracted in RELION-3.1.1 with a box size of 200 pixels and a binning factor of 2. After a reference-free 2D classification by RELION, 452,278 particles were selected for 3D classification using the initial model generated by RELION^36,42^. Selected particles (319,121) were subjected to a 3D auto-refinement. The second round of 3D auto-refinement was performed with *C*2 symmetry and the original pixel size. Then, the selected particles were imported to cryo-SPARC-3.2.0^43^. After ab initio reconstruction, the non-uniform refinement was performed with *C*2 symmetry, resulting in a 2.91 Å resolution map^44^.

Particles for the Fe(III)–DMA–HvYS1 complex were picked using TOPAZ, resulting in a total of 6,213,206 particles. After two rounds of 2D classification by RELION 3.1, 636,852 particles were selected and then imported to cryoSPARC-3.2.0. After *ab* initio reconstruction, a class containing 471,603 particles was subjected to a non-uniform refinement imposing *C*2 symmetry, resulting in a 2.72 Å resolution map.

Finally, the particles For Fe(III)–PDMA–HvYS1 complex were also picked using TOPAZ, resulting in a total of 3,041,199 particles. After two rounds of 2D classification by RELION-3.1.1, 547,619 particles were selected and then imported to cryoSPARC-3.2.0. After *ab* initio reconstruction, a class containing 386,168 particles was subjected to a non-uniform refinement imposing *C*2 symmetry, resulting in a 2.94 Å resolution map.

### Model building and refinement

The atomic model of HvYS1 was manually built using Coot-0.9.8.1^45,46^ from the globally sharpened map at 2.9 Å resolution by cryoSPARC-3.2.0. The apo HvYS1 structure was used as an initial model for the Fe(III)–DMA–HvYS1 complex, and the atomic model was manually adjusted with Coot-0.9.8.1. The Cu(II)–MA structure (The Cambridge Structural Database: 1215044) was used as an initial model of the Fe(III)–DMA complex,^19^. Finally, the HvYS1 structure in the Fe(III)–DMA–HvYS1 complex was used as an initial model of HvYS1 for the Fe(III)–PDMA–HvYS1 complex. The model of PDMA was generated by replacing l-azetidine 2-carboxylic acid of DMA with the proline coordinates. The ligand restraint files were generated by eLBow in Phenix-1.19.2^47,48^. All structural refinements were performed by real-space refinement in Phenix-1.19.2^48,49^. Stereo chemistries for the final models were assessed by MolProbity^50^. The buried surface area was calculated by PISA-2.1.0^51^. The topological similarity within transmembrane domains was analyzed by TMalign^52^. All figures were prepared using PyMol-2.5.2 (https://pymol.org) and UCSF Chimera X-1.2.5^53^.

### Insect cell-based Fe(III)–DMA transport assay

The Fe(III)–DMA transport activity was measured in Sf9 insect cells heterologously expressing HvYS1^7^. A radioactive Fe(III)–DMA complex was prepared as follows. A 100 mM Fe(III) solution was prepared by mixing equal amounts of 180 mM FeCl_3_ and 20 mM 55Fe solution (NEZ043; 37.0 MBq, Perkin-Elmer, Waltham, MA, USA). Then, Fe solution and DMA, dissolved in MES/Tris buffer (pH 6.0), were mixed at an equimolar ratio to be a final concentration of 7.5 mM Fe-DMA (Fe:DMA = 1:2). Undissolved Fe ion was removed by centrifugation prior to use.

A 5 mL of the insect cell culture was harvested 72 h after transfection of the baculovirus by centrifugation at 1700 × *g* for 5 min and resuspended in 1 mL of SF900II medium. Then, 7 μL of 7.5 mM Fe(III)–DMA complex, containing 10% of ^55^Fe, was added to the cell suspension. After gently mixing the cell suspension at room temperature for 1 h, the cells were harvested by centrifugation at 1700 × *g* for 5 min. Then, the cells were subsequently washed three times with 1.0 mL of phosphate-buffered saline (PBS) to remove the residual ^55^Fe in the broth. The cells were lysed in 300 μL of 1 M NaOH and 200 μL of PBS by vortexing. After mixing with 5 ml of liquid scintillation cocktails Ultima Gold^TM^ (PerkinElmer, Waltham, MA, USA), the radioactivity of ^55^Fe in the cell lysate was measured using ALOKA LSC-6100 liquid scintillation counter (Hitachi-Aloka Medical, Tokyo, Japan).

### Immunofluorescence imaging

Surface expression of HvYS1 in the insect cell was confirmed by immunofluorescence imaging (Supplementary Fig. 14). The adherent Sf9 insect cells (1.0 × 10^6^ cells) were infected with the recombinant baculovirus (P2) harboring either wild-type or each mutant *HvYS1* gene in each 35 mm glass dish at 28 °C for 3 days. The Sf9 cells were washed by PBS buffer (pH 7.4) and fixed with 1 mL of 4% paraformaldehyde (PFA, Nacalai tesque) in PBS for 10 min. The cells were permeabilized by 0.2% Triton in PBS at room temperature for 10 min. After washing with PBS three times, the cells were treated with 1% bovine serum albumin (BSA, Sigma) in PBS for blocking at room temperature for 20 min. The cells were reacted with anti-HvYS1 polyclonal antibodies (Rabbit, 1:200 dilution)^12^ at 4 °C overnight. After washing three times with PBS, the cells were reacted with anti-rabbit secondary antibody conjugated with Alexa 546 (Thermo Fisher, # A10040; Donkey, 1:1000 dilution) at room temperature for 1 h. For double staining after blocking, the cells reacted with anti-the plasma membrane Ca^2+^ ATPase (PMCA) antibodies (Thermofisher #MA3914; Mouse, 1:200 dilution) at 4 °C for overnight. After washing three times with PBS, the cells were reacted with anti-mouse secondary antibody conjugated with Alexa 488 (Thermofisher # A11029; Goat, 1:1000 dilution) at room temperature for 1 h. After washing three times with PBS, the cells were examined with a laser scanning confocal microscope (Olympus FV3000).

In addition, to confirm the similar expression level and the proper folding of each mutant protein, the wild-type or the mutant HvYS1 was purified from 1 L of the Sf9 cultured cells in the manner described above. The fractions after Ni-affinity purification and the pooled fractions of the HvYS1 dimer in SEC were analyzed by SDS-PAGE with CBB-staining (Supplementary Fig. 15). The proper folding of each protein was confirmed by SEC profiles (Supplementary Fig. 15). The uncropped gel images are shown in Supplementary Fig. 16.

### Molecular dynamics (MD) simulations

Molecular dynamics simulations were performed for twelve systems with different protonation states of Asp446, Asp490, Asp494, and Asp651. Since Asp651 is in the scaffold domain and unlikely to be involved in the protonation for the transport, the results of the simulations with Asp651 protonated were omitted for clarity. On the assumption that the change of the protonation state of one chain would not affect the structure of the other chain, different protonation states were assigned to the two chains in each system (Supplementary Table 2). In both the chains of all the systems, Asp270 and Asp617 were protonated, while the remaining aspartate and glutamate residues were deprotonated. Histidine residues were protonated on the Nε2 atoms, except for His344, which forms a salt bridge with Asp340, whereby it was protonated on both the Nδ1 and the Nε2 atoms. Each system was constructed in essentially the same manner. The orientation and the position of the dimeric structure in a lipid bilayer were predicted by the Positioning of Proteins in Membrane (PPM) server^54^. The dimeric structure was then embedded in a solvated lipid bilayer using the “Membrane Builder” function^55^ of the CHARMM-GUI server^56^. The system was composed of two protein chains, 2 Fe(III)–DMA, four cholesterols in replacement of CHS, and 614 1-palmitoyl-2-oleoyl-*sn*-glycero-3-phosphocholine (POPC) molecules, ~160 K^+^ and 210 Cl^−^ ions, and 59,600 water molecules. In our cryo-EM structure of HvYS1 in complex with Fe(III)–DMA, the Fe^3+^ ion is coordinated by four oxygen atoms (O1, O4, O6, and O8) and two nitrogen atoms (N1 and N2) of DMA. We imposed harmonic restraints on the distances between the Fe^3+^ ion and the ligand atoms with a force constant of 1.0 × 10^5 ^kJ mol^−1^ nm^−2^ and with equilibrium distances of 2.0 Å for the oxygen ligand atoms and of 2.5 Å for the nitrogen ligand atoms. Therefore, the Fe^3+^–ligand distances were maintained during the simulations. The size of the initial system was 16.0 nm × 16.0 nm × 11.7 nm. The topology and the parameters of DMA were generated using the “Ligand Reader & Modeler” function^57^ of the CHARMM-GUI server and the CHARMM general force field (CGenFF)^58^. The atomic charges of DMA and Fe^3+^ were replaced with the quantum-mechanically calculated values. Geometry optimization and the electrostatic potential calculation of Fe(III)–DMA were performed using Gaussian 16, Revision B.01^59^, with the Hartree-Fock method and with the Los Alamos National Laboratory 2 double zeta (LANL2DZ) basis set^60^ for the iron atom and the 6–31 G(d) basis set for the other atoms. The restrained electrostatic potential (RESP) method^61^ was used to determine the atomic charges from the electrostatic potential. The TIP3P model^62^ was used for the water molecules. The CHARMM36m force field^63^ was used for the protein chains and the CHARMM36 force field^58,64^ was used for the other molecules. After the system was energy-minimized, it was equilibrated in nine steps. In the first two steps, the system was equilibrated for 0.25 ns in the *NVT* ensemble, and in the following three steps, it was equilibrated for 1.125 ns in the *NPT* ensembles. In all these steps, the positions of the protein and the ligand non-hydrogen atoms were restrained to their initial positions. The force constants were gradually decreased from 4000 to 200 kJ nm^−2^ for the protein backbone and the ligand atoms, and from 2000 to 50 kJ nm^−2^ for the protein sidechain atoms. The Z coordinates of the phosphorus atoms of the POPC molecules were also restrained with force constants from 1000 to 40 kJ nm^−2^. The distances between the protein and the ligand atoms that form intermolecular hydrogen bonds were restrained with force constant of 4000 kJ nm^−2^. In the sixth step, a 0.5-ns MD simulation was performed with position restraints of 50 kJ nm^−2^ imposed only on the protein backbone atoms and with distance restraints of 4000 kJ nm^−2^. No restraints were imposed on the lipid atoms. In the last three steps, MD simulations were performed for 100 ns in total, only with the distance restraints. The force constant was decreased from 4000 kJ nm^−2^ to 200 kJ nm^−2^. Finally, a 500-ns production MD simulation was performed without restraints. The temperature was kept at 303.15 K throughout the MD simulations and the pressure was kept at 1.0 × 10^5 ^Pa, except for the first two equilibration steps. In the equilibration steps, the Berendsen weak coupling method^65^ was used to control the temperature and the pressure, and in the production run, the Nosé-Hoover method^66,67^ and the Parrinello-Rahman method^68,69^ were used to control each, respectively. Bond lengths involving hydrogen atoms were constrained using the LINCS algorithm^70,71^ to allow the use of a large time step (2 fs). Electrostatic interactions were calculated with the particle mesh Ewald method^72,73^. All the MD simulations were performed using GROMACS 2021^71^, with coordinates recorded every 10 ps. Principal component analysis (PCA) was performed to investigate the conformational changes of the protein during the MD simulations^74^. In this analysis, only the coordinates of the Cα atoms in all the helical segments (Fig. 1f) were used. The average coordinates and the principal axes were calculated from the combined trajectory of the twelve systems.

### Reporting summary

Further information on research design is available in the Nature Portfolio Reporting Summary linked to this article.

## Supplementary information

Supplementary Information

Peer Review File

Reporting Summary

## Data Availability

The cryo-EM maps have been deposited into the Electron Microscopy Data Bank under accession numbers EMD-32765 (apo), EMD-32766 (with Fe(III)–DMA), EMD-32767 (with Fe(III)–PDMA). The coordinates have been in the RCSB Protein Data Bank (PDB) accession codes 7WSR (apo), 7WST (with Fe(III)–DMA), and 7WSU (with Fe(III)–PDMA), respectively. The source data underlying Figs. 3f and 5f are provided as a Source Data file. Source data are provided with this paper.

## Source data

Source Data
