# Supplementary material for: Uptake mechanism of iron-phytosiderophore from the soil based on the structure of yellow stripe transporter
Source: Nat Commun. 2022 Nov 23;13:7180. doi: 10.1038/s41467-022-34930-1 (PMC9691689; doi:10.1038/s41467-022-34930-1)
Supplement: Supplementary file 1 — Supplementary Information [file 41467_2022_34930_MOESM1_ESM.pdf]

Supplementary Information

Uptake mechanism of iron-phytosiderophore from the soil based on the structure of Yellow Stripe transporter

Atsushi Yamagata, Yoshiko Murata, Kosuke Namba, Tohru Terada, Shuya Fukai, Mikako Shirouzu

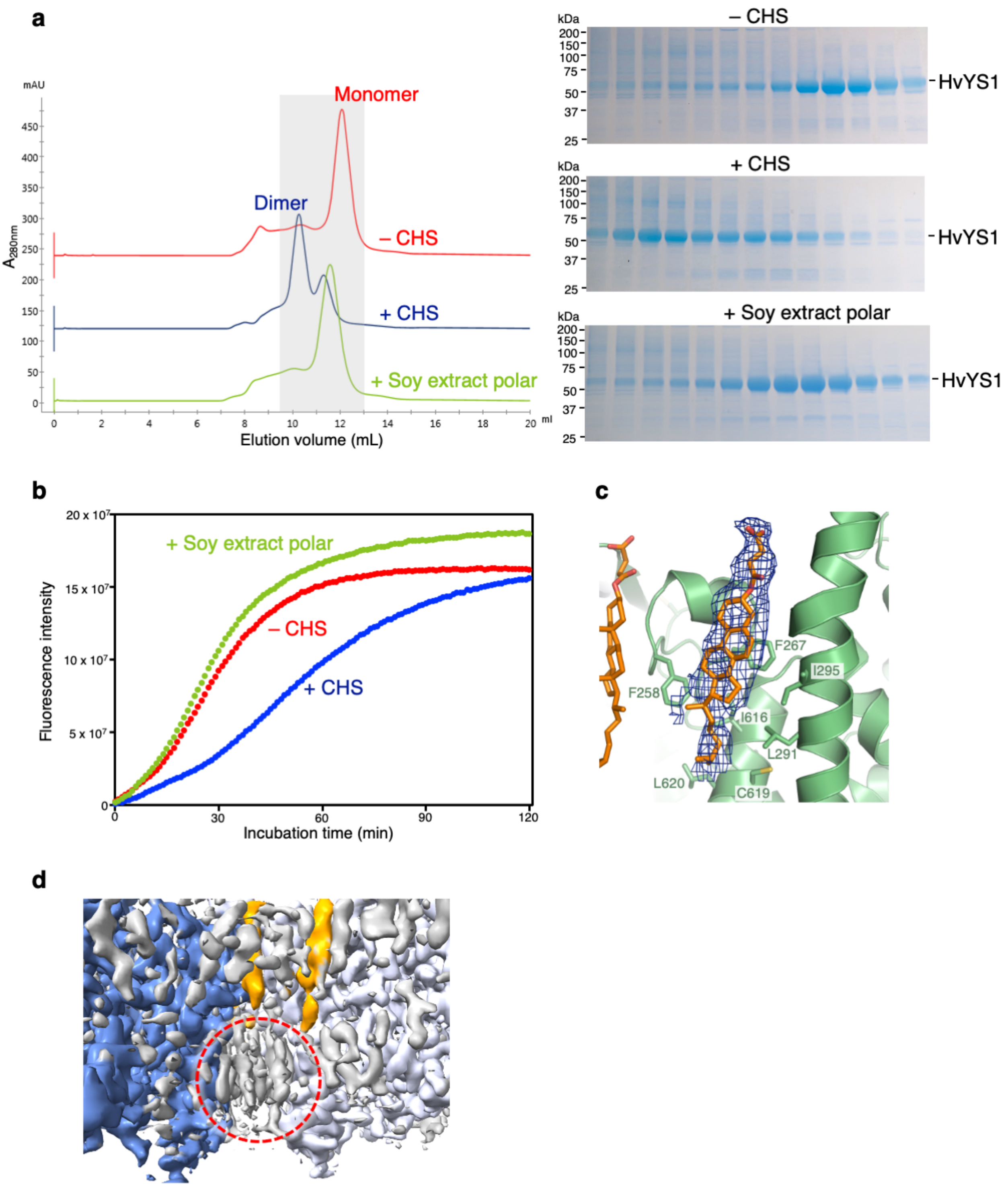

Supplementary Figure 1. The dimer formation and the enhanced thermal stability of HvYS1 by CHS.

a. Size exclusion chromatography (SEC) profiles of HvYS1 with CHS (+CHS; blue), without additional lipid (–CHS; red), and with soy extract polar lipid (green) after Ni-affinity purification. The corresponding SDS-PAGE gels of the shadowed area are shown right. Experiments were repeated twice independently with similar results. **b.** Fluorescence-based thermal stability assay. The fluorescence of CPM is enhanced by binding to cysteine residues exposed during heat denaturation at 40 °C. **c.** The second CHS molecule bound within HvYS1 subunit. **d.** Cryo-EM density map near the dimer interface. The bound CHS molecules are highlighted in orange. The additional lipid-like densities at the cytoplasmic part of the two subunits interface are indicated by red circle.

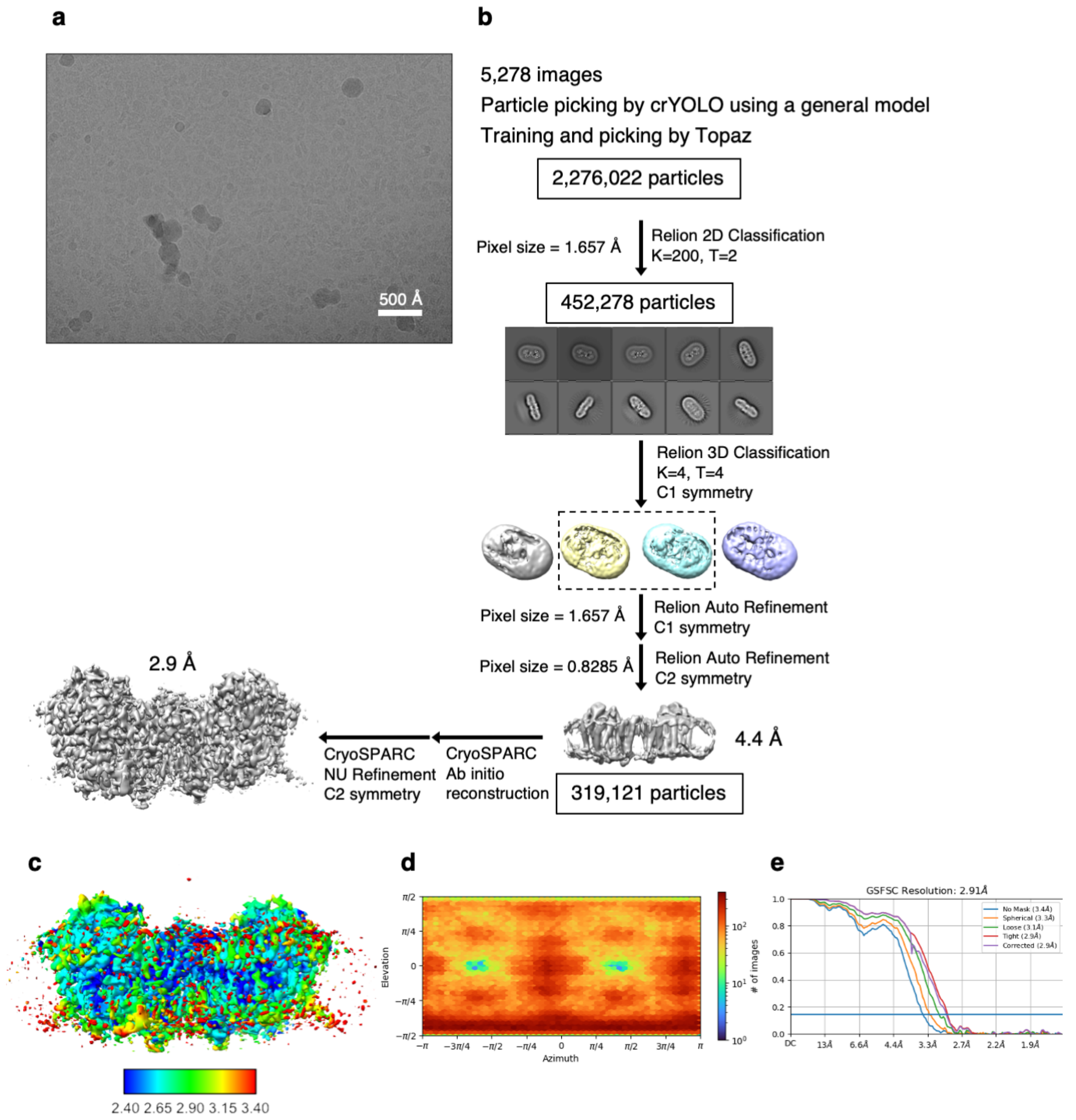

Supplementary Figure 2. Cryo-EM data and processing for HvYS1 in the apo state.

a. A representative micrograph of HvYS1. Experiments were repeated twice independently with similar results. **b.** A flow chart for data processing and the final map of HvYS1. **c.** Local-resolution map of HvYS1. **d.** Euler angle distribution plot of the final 3D reconstruction of HvYS1. **e.** Fourier shell correlation (FSC) curves for HvYS1.

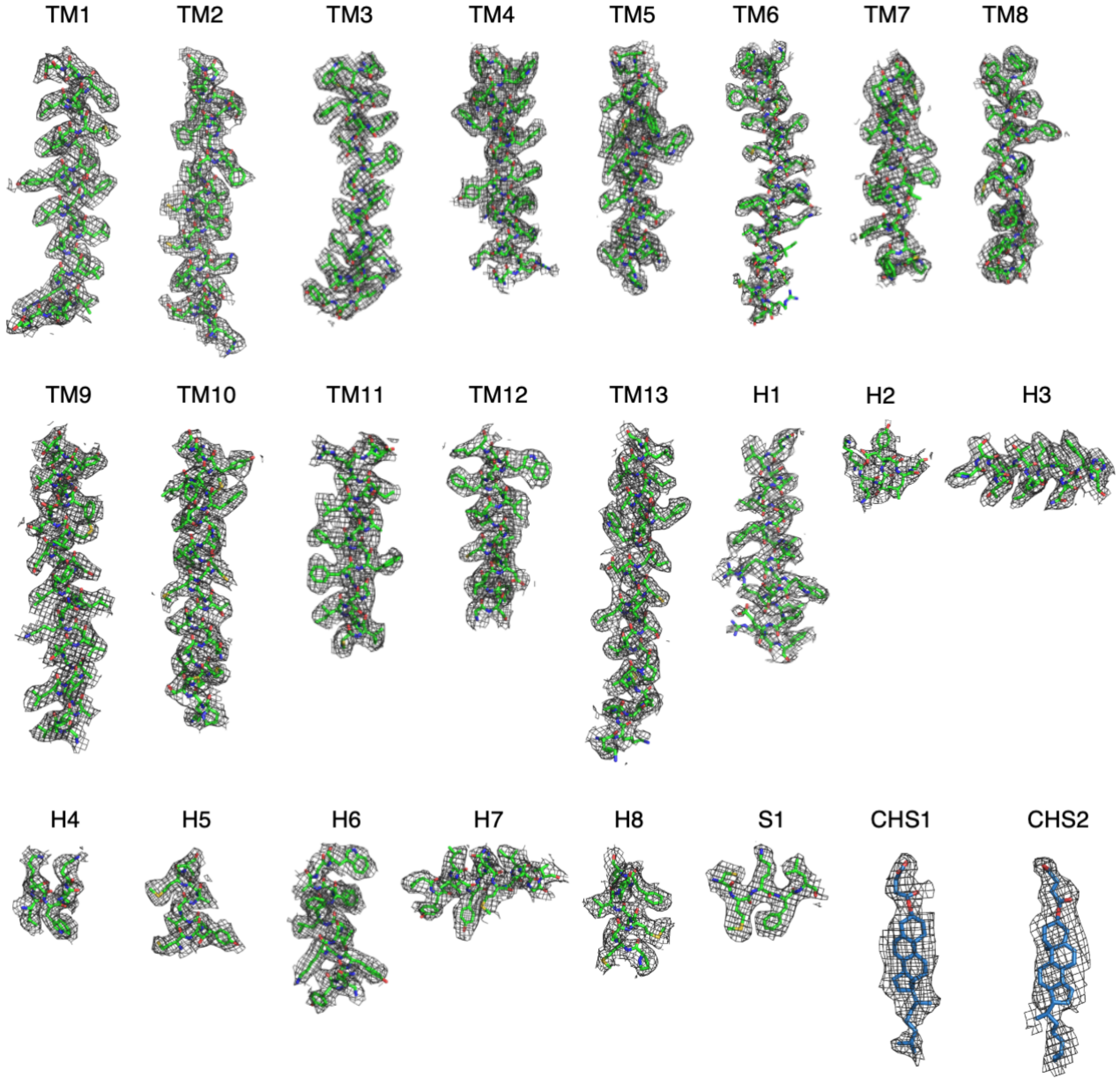

Supplementary Figure 3. Density maps for HvYS1 in the apo state.

Cryo-EM densities for HvYS1 in the apo state superimposed onto the model.

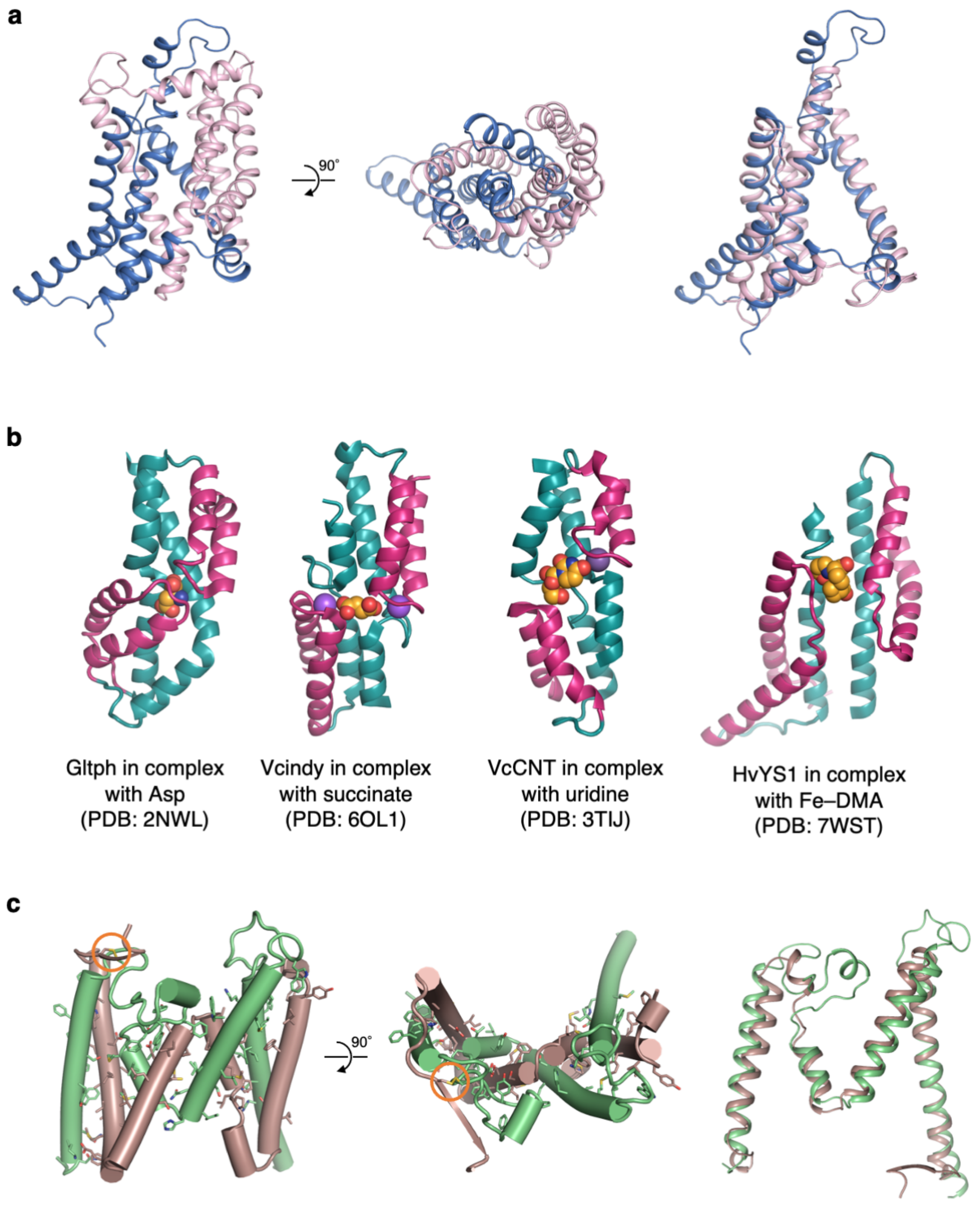

Supplementary Figure 4. Symmetry-related inverted repeats

a. The YS core domain (left, middle) and the superposition of N- and C-halves (right) are related as an inverted pseudo symmetry. N- and C-halves are colored in blue and pink, respectively. **b.** The repeats of the helix hairpins and the subsequent central TM comprise the substrate-binding interface and ion-binding site in elevator-like transporters. **c.** The scaffold domain (left, middle) and the superposition of N- and C-halves (right) are related as an inverted pseudo symmetry. N- and C-halves are colored in green and brown, respectively. The disulfide bond is shown in sticks and highlighted in an orange circle. The residues involved in the interaction between two halves are shown in sticks.

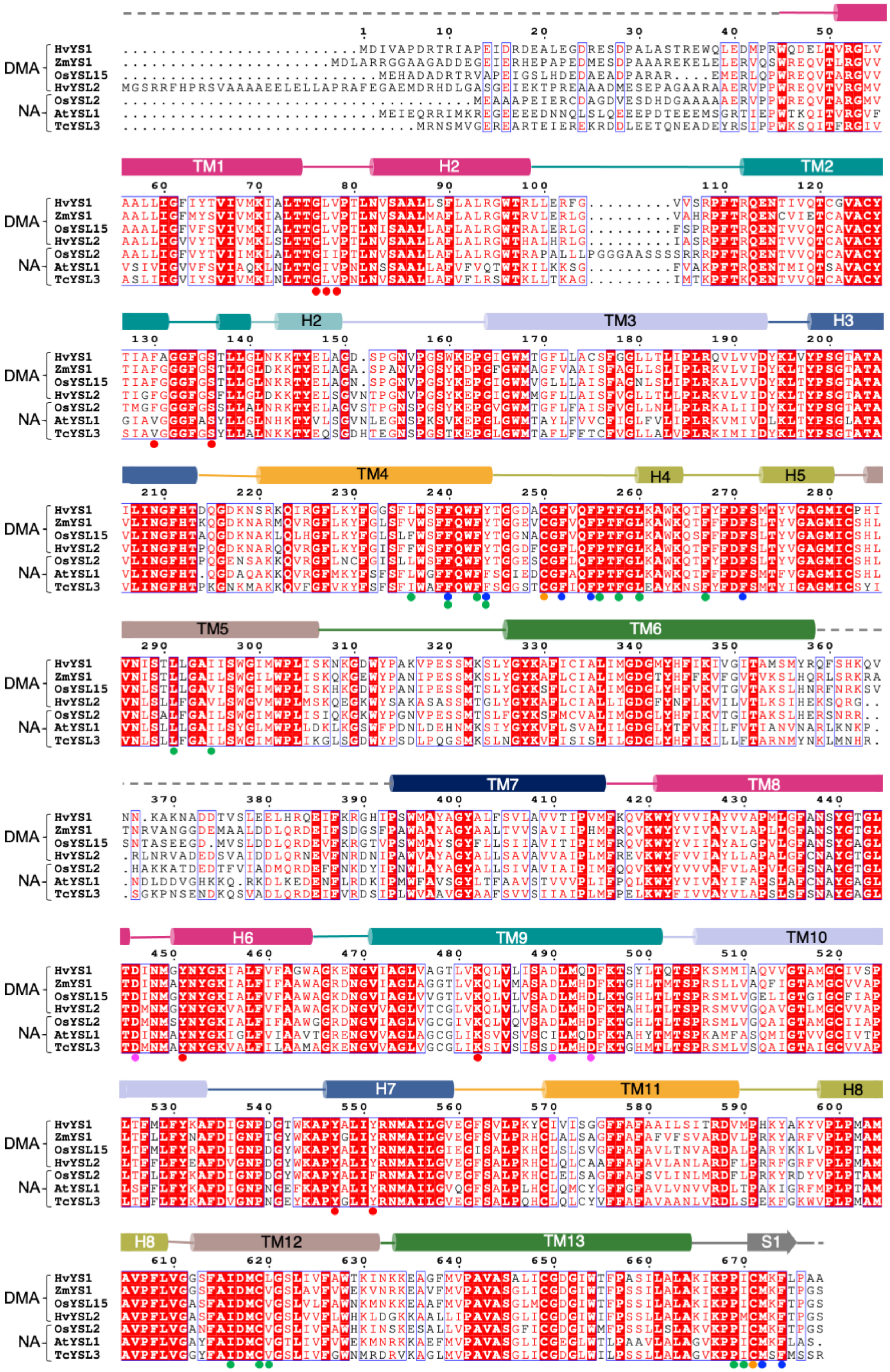

DMA
NA
HvYS1
ZmYS1
OsYSL15
HvYSL2
OsYSL2
AtYSL1
TcYSL3
TM1
H2
TM2
H3
TM3
TM4
H4
H5
TM5
TM6
TM7
TM8
H6
TM9
TM10
H7
TM11
H8
TM12
TM13
S1

Supplementary Figure 5. Multiple sequence alignment.

Barley YS1 (*Hordeum vulgare L.* YS1, HvYS1; accession No. BAE71187), Maize YS1 (*Zea mays* YS1, ZmYS1; accession No. Q9AY27), Rice YSL15 (*Oryza sativa* YSL15, OsYSL15; accession No. Q6H3Z3), Barley YSL2 (*Hordeum vulgare L.* YSL2, HvYSL2; accession No. BAL02122), rice YSL2 (*Oryza sativa* YSL2, OsYSL2; accession No. Q6H3Z6), *Arabidopsis thaliana* YSL1 (AtYSL1: accession No. Q6R3L0), and *Thlaspi caerulescens* YSL3 (TcYSL3; accession No. DQ268829) are aligned using ClustalW[1] and displayed with Espript3.0[2]. The former four proteins transport metal–DMA complex, whereas the latter three proteins transport metal–NA complex. Secondary structural elements are shown above. The residues involved in the interaction with Fe(III)–DMA are highlighted in red circles. The dimer interface residues and two cysteine residues forming a disulfide bond are marked by blue and orange circles, respectively. The residues involved in the interaction with CHS are marked by green circles. The potential proton binding sites, Asp446, Asp490, and Asp494 are marked by a magenta circle.

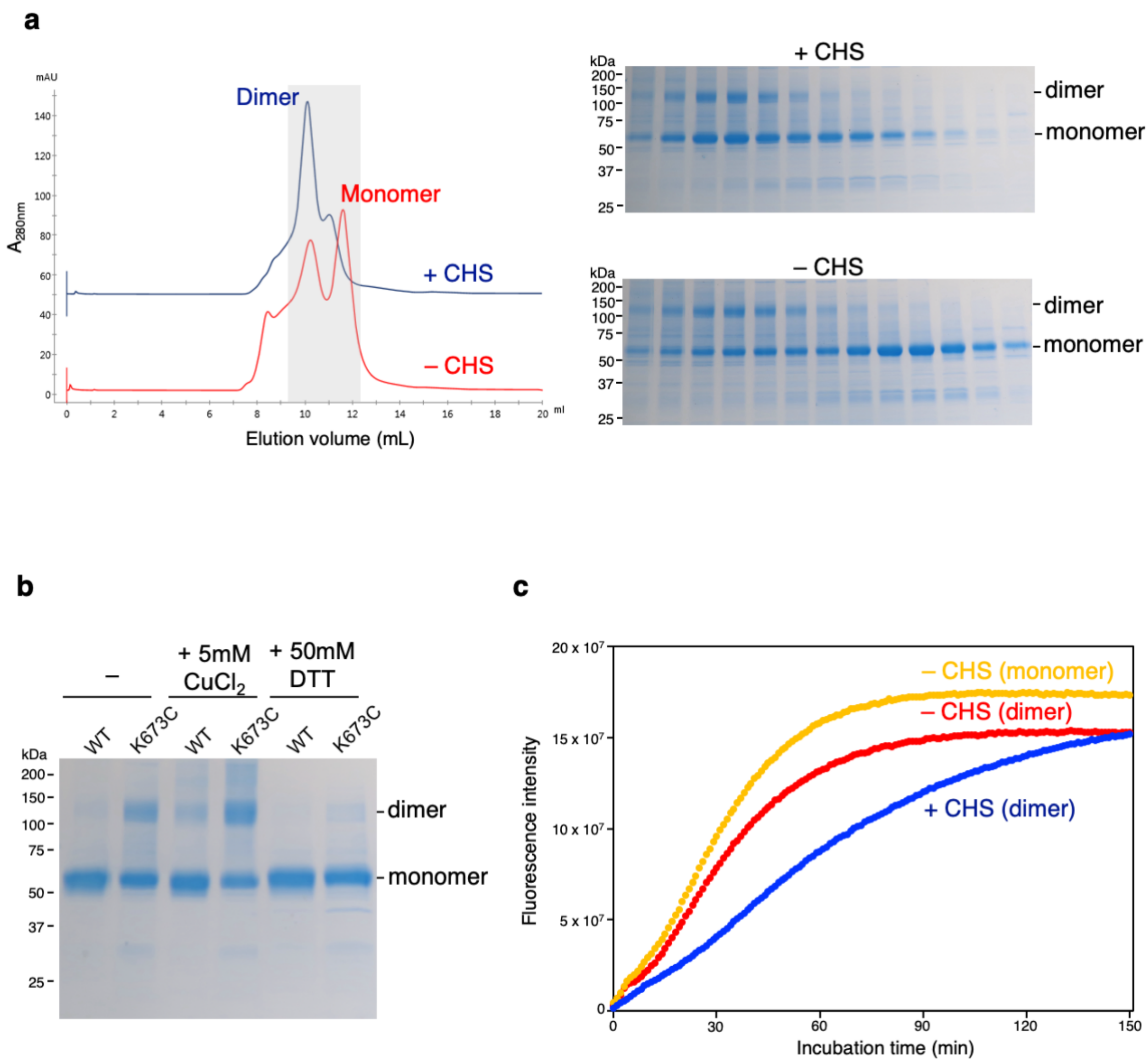

Supplementary Figure 6. The disulfide crosslinked dimer of HvYS1^{K673C}.

a. SEC profiles of HvYS1^{K673C} with (blue) and without CHS (red) after Ni-affinity purification. The corresponding non-reducing SDS-PAGE gels of the shadowed area are shown right. The spontaneously formed crosslinked dimer was detected. Experiments were repeated twice independently with similar results. **b.** Enhancement of the crosslinked dimer by $CuCl_2$ and reduction by DTT. The non-reducing SDS-PAGE of the purified HvYS1^{wt} (lane 1 from left) and HvYS1^{K673C} (lane 2). The spontaneously formed crosslinked dimer was observed in HvYS1^{K673C}. The crosslinked dimer of HvYS1^{K673C} was enhanced by the addition of 5 mM of $CuCl_2$, an oxidant (lane4). The treatment with 50 mM DTT almost completely reversed the crosslinked dimer of HvYS1^{K673C} (lane 6). Experiments were repeated twice independently with similar results. **c.** Fluorescence-based thermal stability assay of HvYS1^{K673C}. In the absence of CHS, the dimeric HvYS1^{K673C} does not show higher thermal stability than that of the monomeric HvYS1^{K673C}.

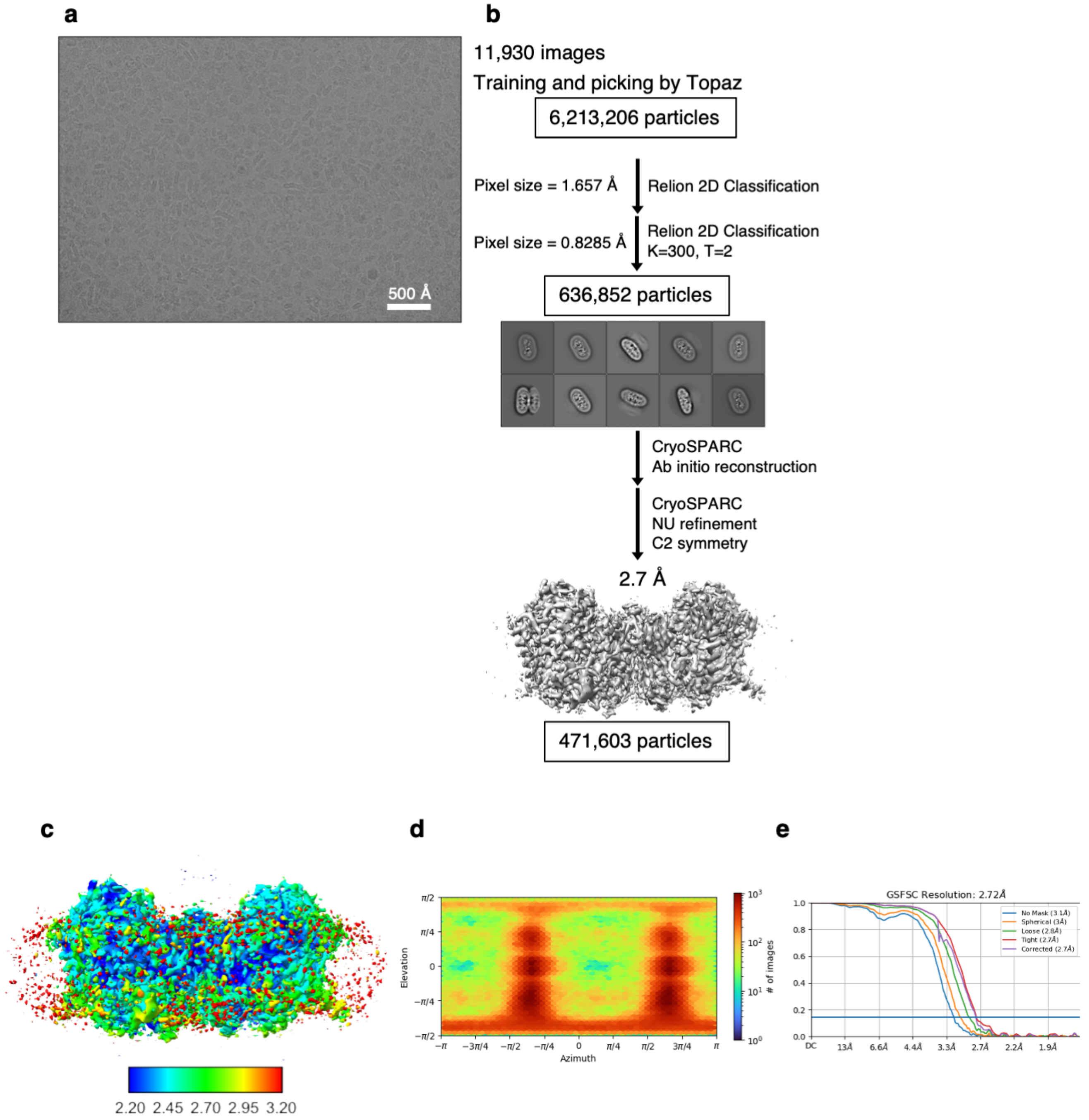

Supplementary Figure 7. Cryo-EM data and processing for HvYS1 in complex with Fe(III)–DMA.

a. A representative micrograph of HvYS1 in complex with Fe(III)–DMA. We made at least two vitrified grids with similar particle images, and the micrographs were collected from a single grid. **b.** A flow chart for data processing and the final map of HvYS1 in complex with Fe(III)–DMA. **c.** Local-resolution map of HvYS1 in complex with Fe(III)–DMA. **d.** Euler angle distribution plot of the final 3D reconstruction of HvYS1 in complex with Fe(III)–DMA. **e.** Fourier shell correlation (FSC) curves for HvYS1 in complex with Fe(III)–DMA.

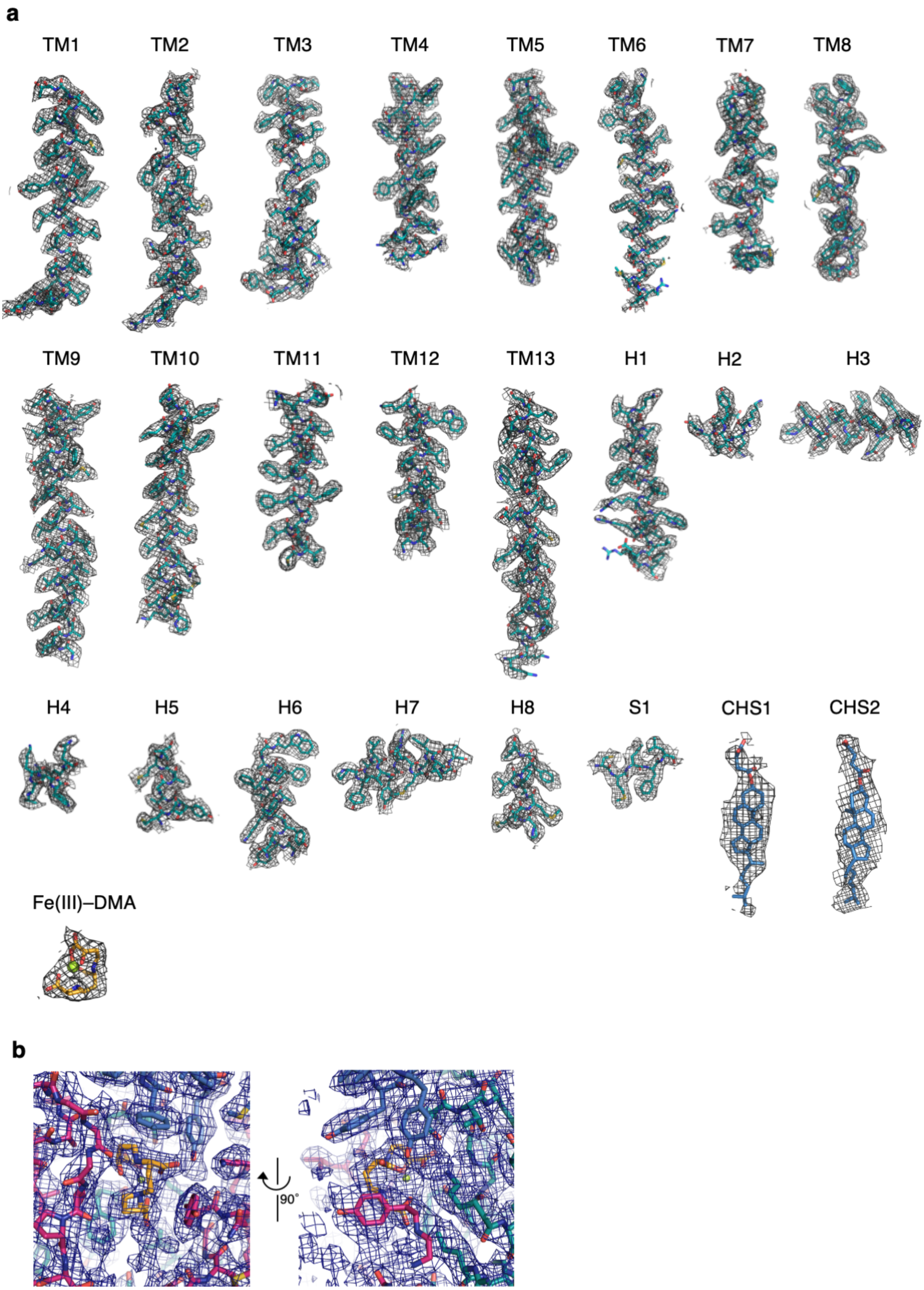

Supplementary Figure 8. Density maps for HvYS1 in complex with Fe(III)–DMA.

a. Cryo-EM densities for HvYS1 in complex with Fe(III)–DMA superimposed onto the model. **b.** Cryo-EM densities for Fe(III)–DMA with the surrounding residues, viewed from two different angles.

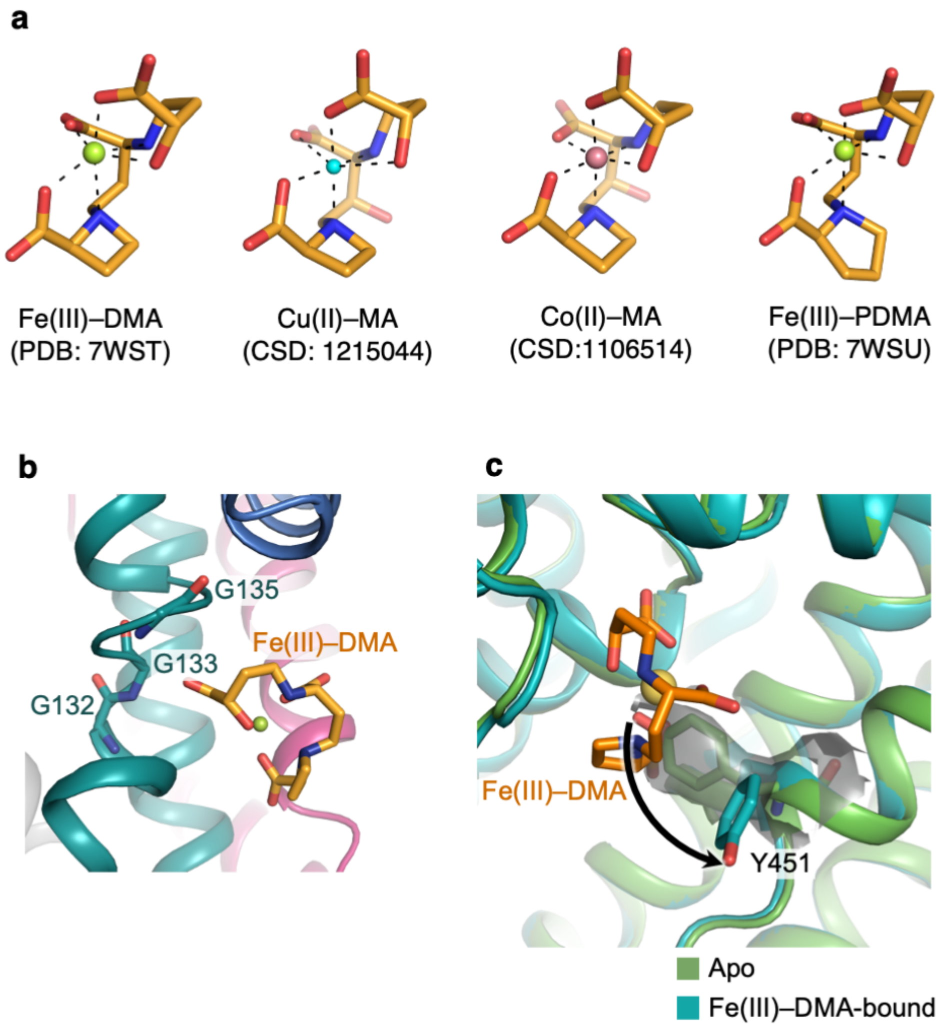

Supplementary Figure 9. Substrate binding site structures of HvYS1

a. The octahedral coordination sphere of metal in the Fe(III)–DMA bound to HvYS1, Cu(II)–MA, Co(II)–MA, and the Fe(III)–PDMA bound to HvYS1. **b.** Side view of the Fe(III)–DMA binding site. Fe(III)–DMA is bound to the unwound part of TM2. **c.** The apo HvYS1 structure is superposed onto the Fe(III)–DMA bound HvYS1 structure. The density of Tyr451 in the apo HvYS1 structure, contoured at 5σ, is superposed onto the model.

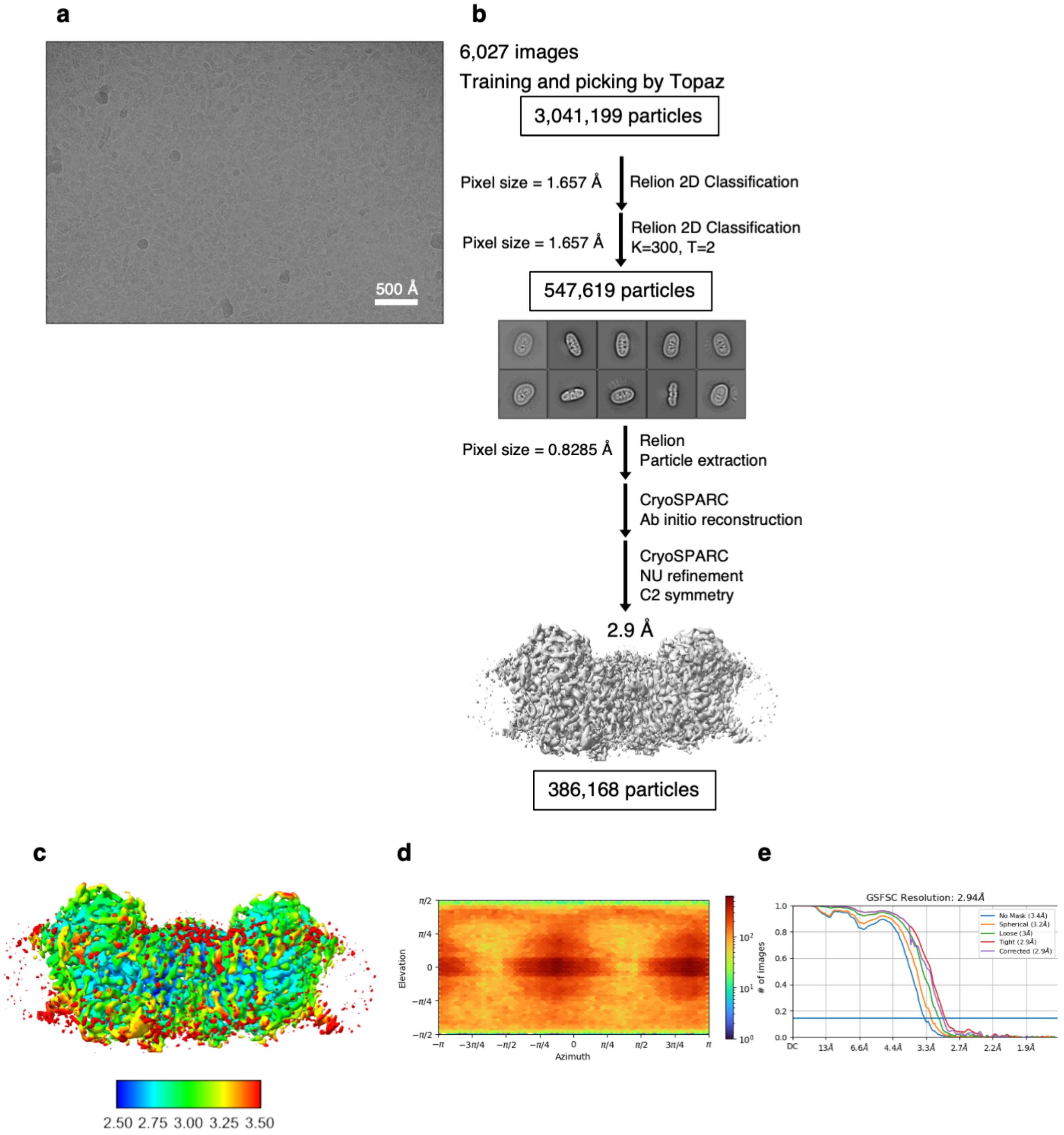

Supplementary Figure 10. Cryo-EM data and processing for HvYS1 in complex with Fe(III)–PDMA.

a. A representative micrograph of HvYS1 in complex with Fe(III)–PDMA. We made at least two vitrified grids with similar particle images, and the micrographs were collected from a single grid. **b.** A flow chart for data processing and the final map of HvYS1 in complex with Fe(III)–PDMA. **c.** Local-resolution map of HvYS1 in complex with Fe(III)–PDMA. **d.** Euler angle distribution plot of the final 3D reconstruction of HvYS1 in complex with Fe(III)–PDMA. **e.** Fourier shell correlation (FSC) curves for HvYS1 in complex with Fe(III)–PDMA.

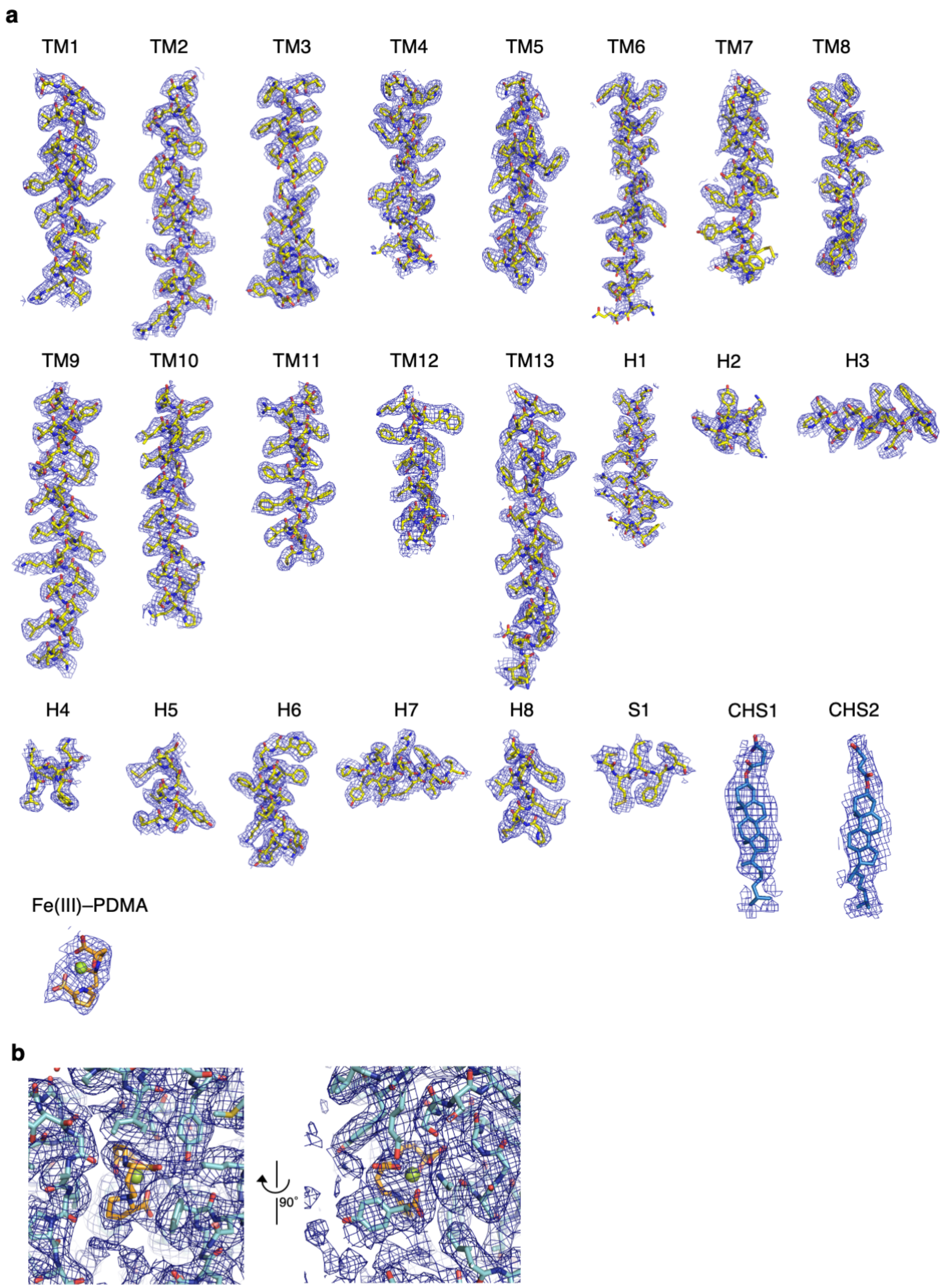

Supplementary Figure 11. Density maps for HvYS1 in complex with Fe(III)–PDMA.

a. Cryo-EM densities for HvYS1 in complex with Fe(III)–PDMA superimposed onto the model. **b.** Cryo-EM densities for Fe(III)–PDMA with the surrounding residues, viewed from two different angles.

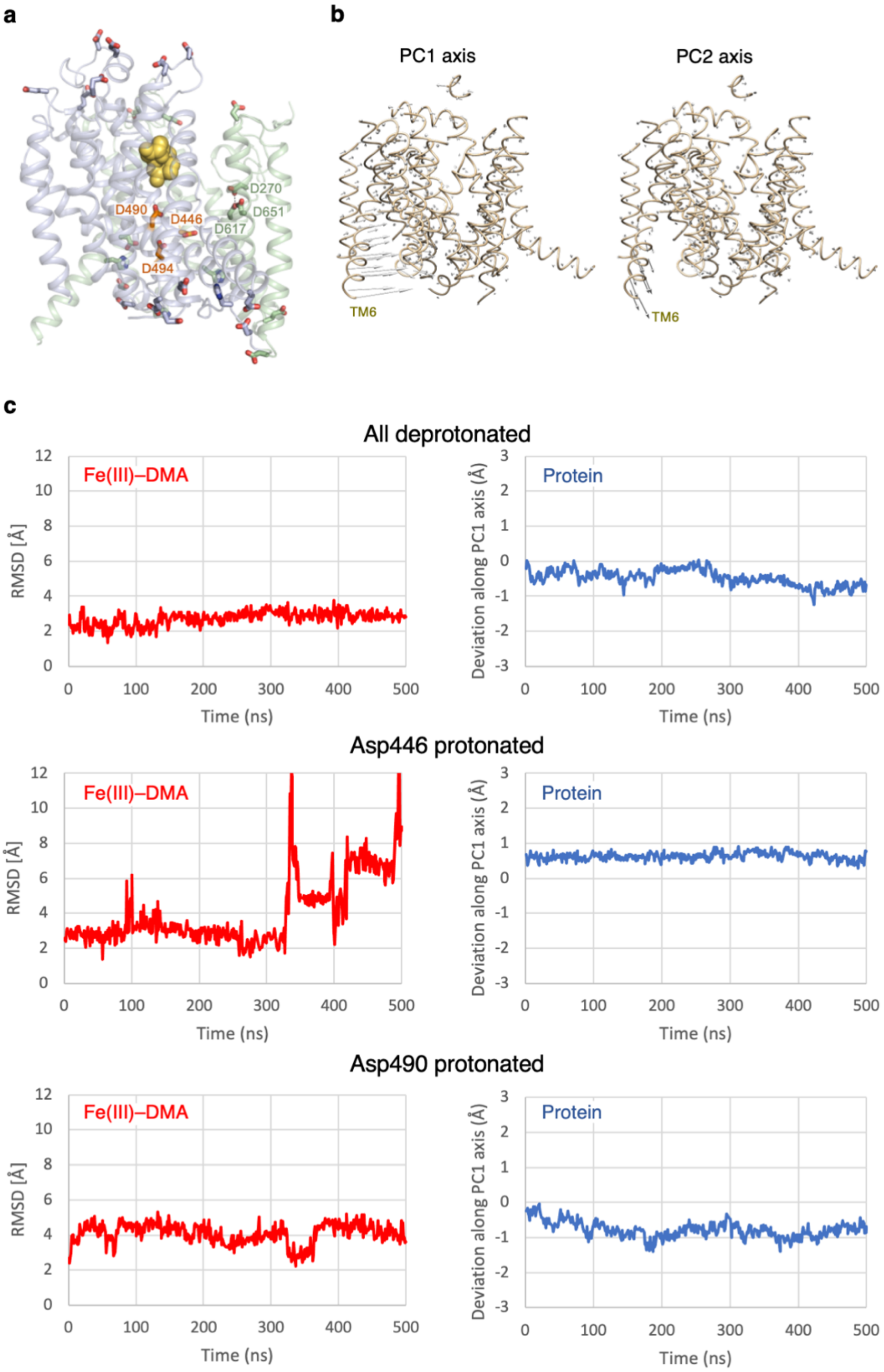
a
D270
D490
D446
D651
D617
D494
b
PC1 axis
PC2 axis
TM6
TM6
c
All deprotonated
Fe(III)–DMA
RMSD [Å]
Time (ns)
Protein
Deviation along PC1 axis (Å)
Time (ns)
Asp446 protonated
Fe(III)–DMA
RMSD [Å]
Time (ns)
Protein
Deviation along PC1 axis (Å)
Time (ns)
Asp490 protonated
Fe(III)–DMA
RMSD [Å]
Time (ns)
Protein
Deviation along PC1 axis (Å)
Time (ns)

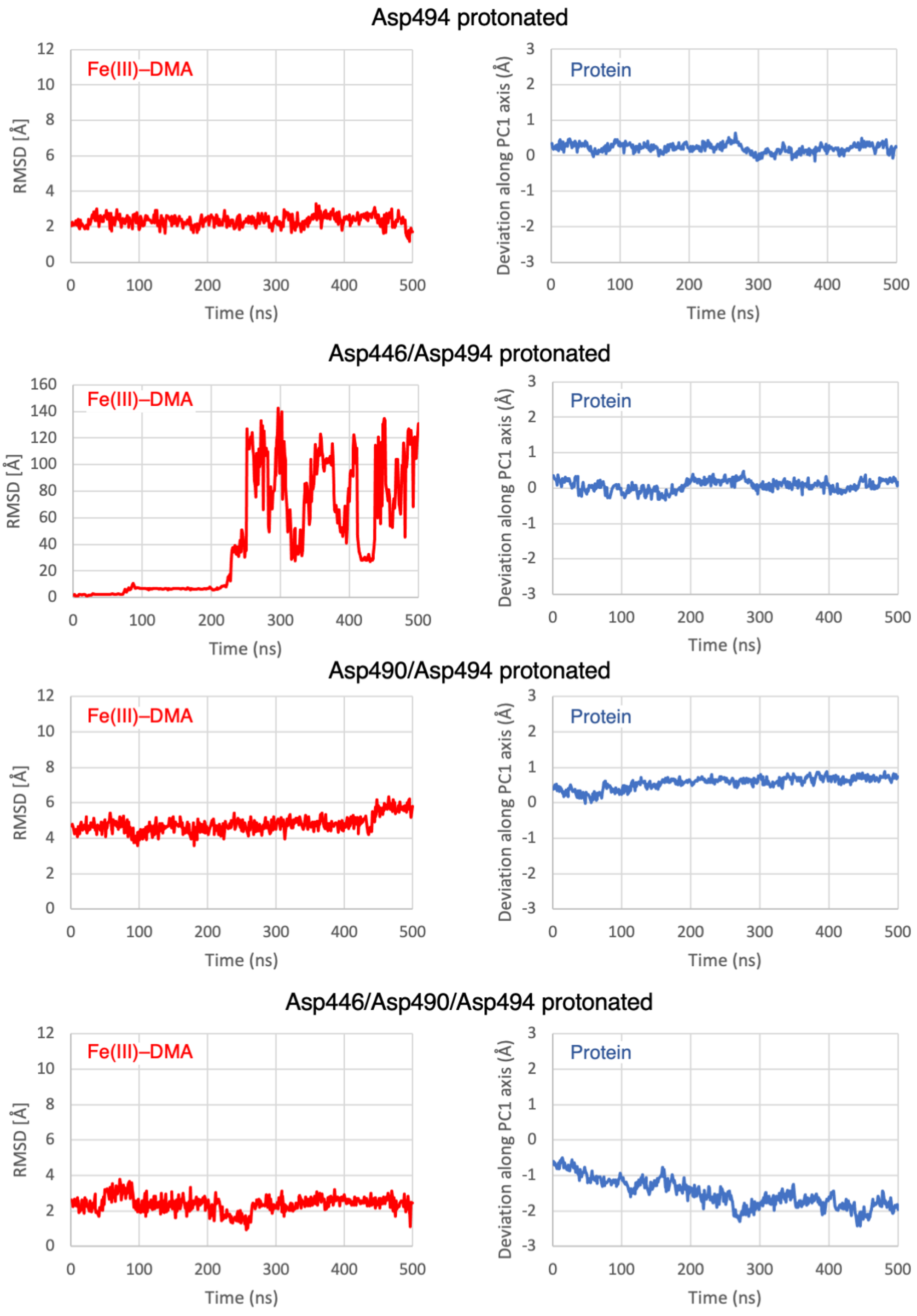

Supplementary Figure 12. MD simulation for each protonated state

a. Asp, Glu, and His residues in HvYS1, shown in sticks. Among them, six Asp residues are on the surface of the central cavity, and three of them (orange) are in the YS core, likely to be the potential proton-binding sites. **b.** Principal component analysis of MD simulations. The two largest deviations (PC1 and PC2) mainly occur on TM6. **c.** The rmsd of the Fe(III)–DMA (left, red) and the deviation along the first principal axis (PC1) (right, blue) of the protein in MD simulations. The ligand rmsd was calculated for non-hydrogen atoms from the initial structure after the protein Cα atoms of the MD structure were aligned to those of the initial structure. In the simulations with Asp446 protonated and those with Asp446/Asp494 protonated, Fe(III)–DMA is released from the binding site.

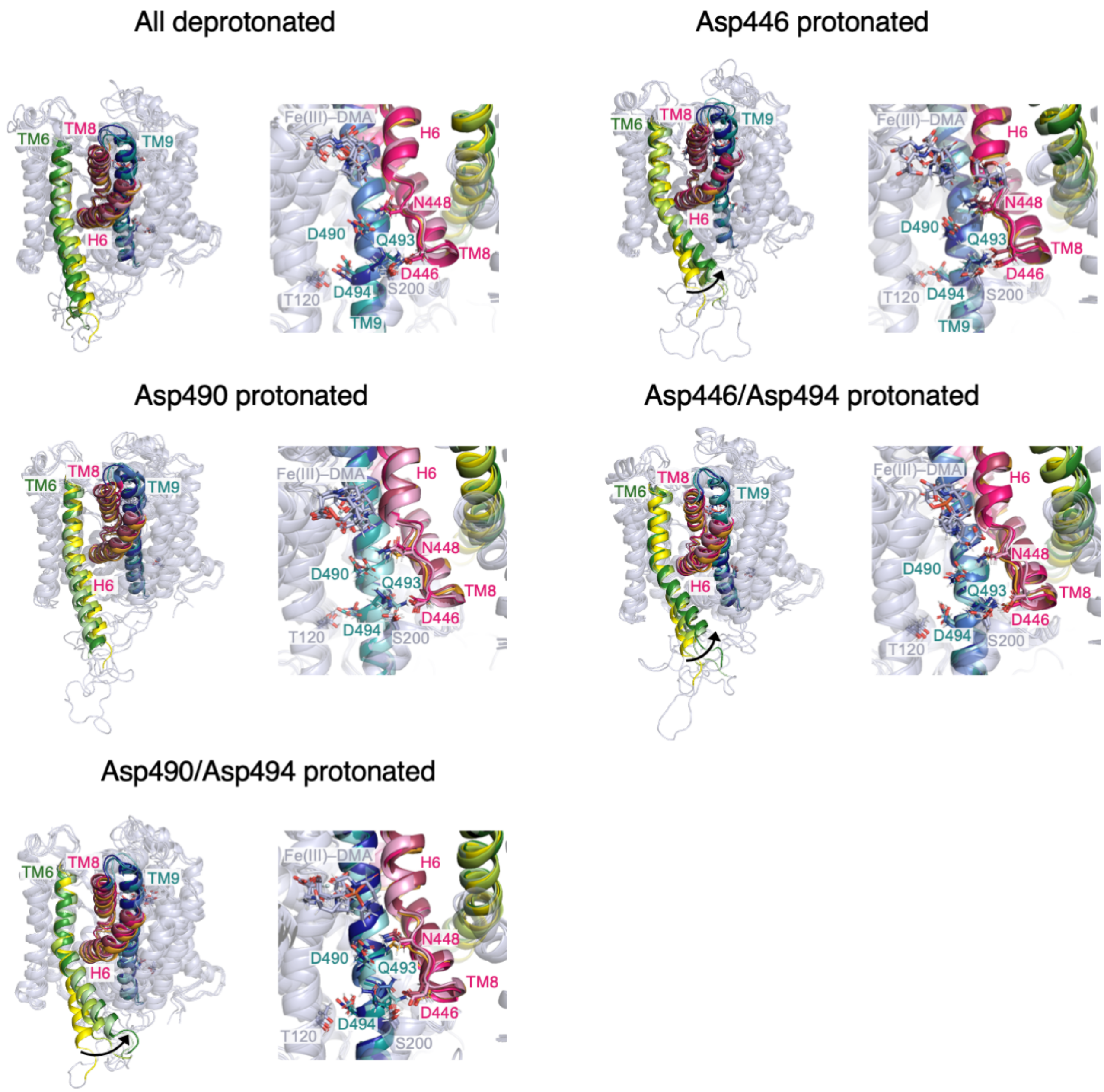

Supplementary Figure 13. MD simulations of HvYS1 in complex with Fe(III)–DMA

The fluctuation of TM6, TM8–H6 in MD simulations are shown in the right. Note that the TM6 approaches the YS core in the simulations with Asp446 protonated, Asp446/Asp494 protonated, and Asp490/Asp494 protonated, in contrast to that with Asp446/Asp490/Asp494 protonated (Fig. 5c). The interhelix H-bonds with three Asp residues and their conformations in MD simulations are shown in the right.

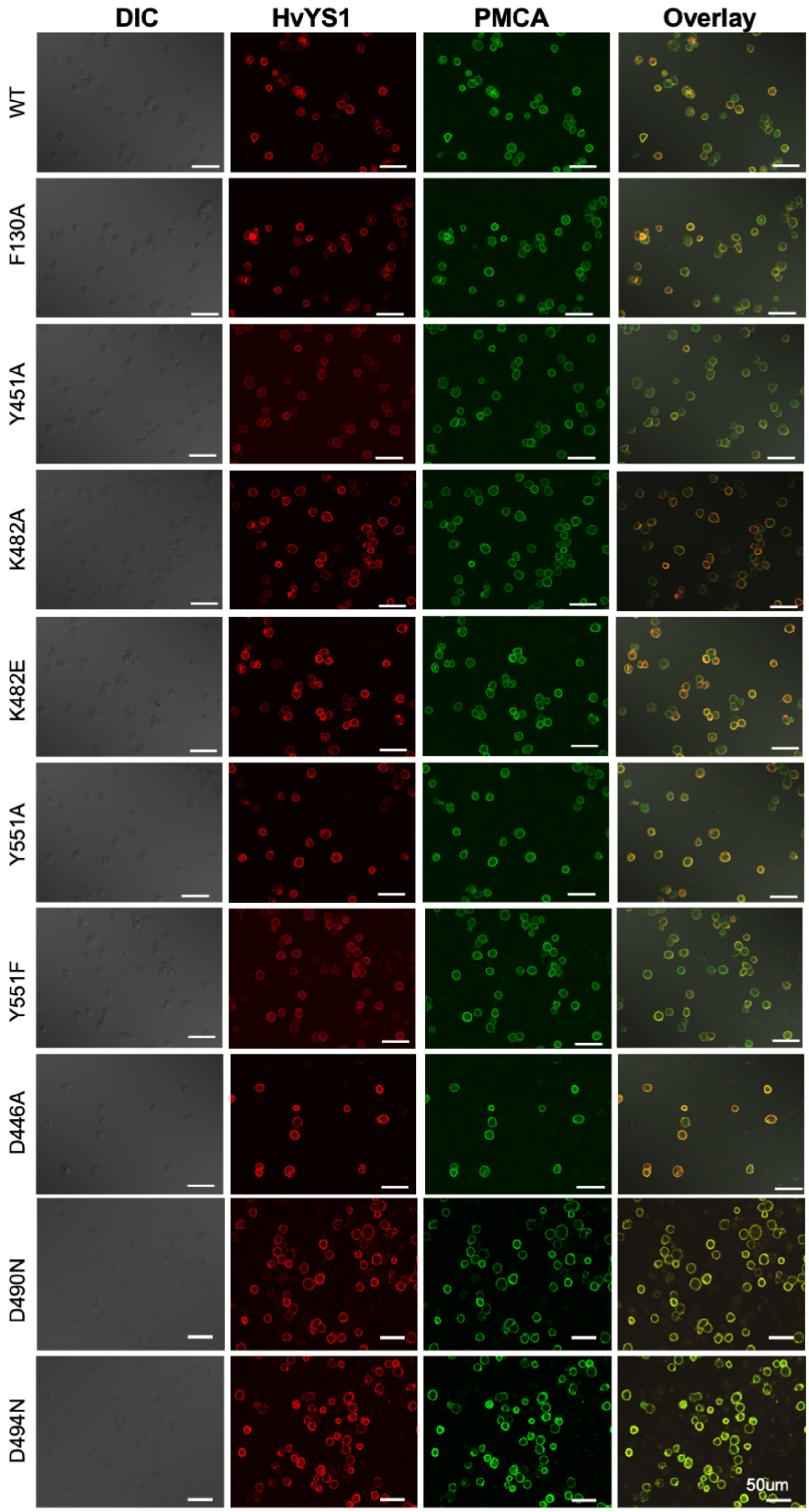

Supplementary Figure 14. Surface expression of the wild-type and mutant HvYS1s

Insect cells expressing HvYS1-wild type or mutants were immunohistochemically visualized by the staining with anti-HvYS1 polyclonal antibody (HvYS1, red), simultaneous immunostaining with anti-the plasma membrane Ca^{2+} ATPase antibody (PMCA, green) was performed. DIC, differential interference contrast. Scale bar indicates 50 μm. Each image is a representative image from three images for each WT or mutant, and two independent experiments were performed.

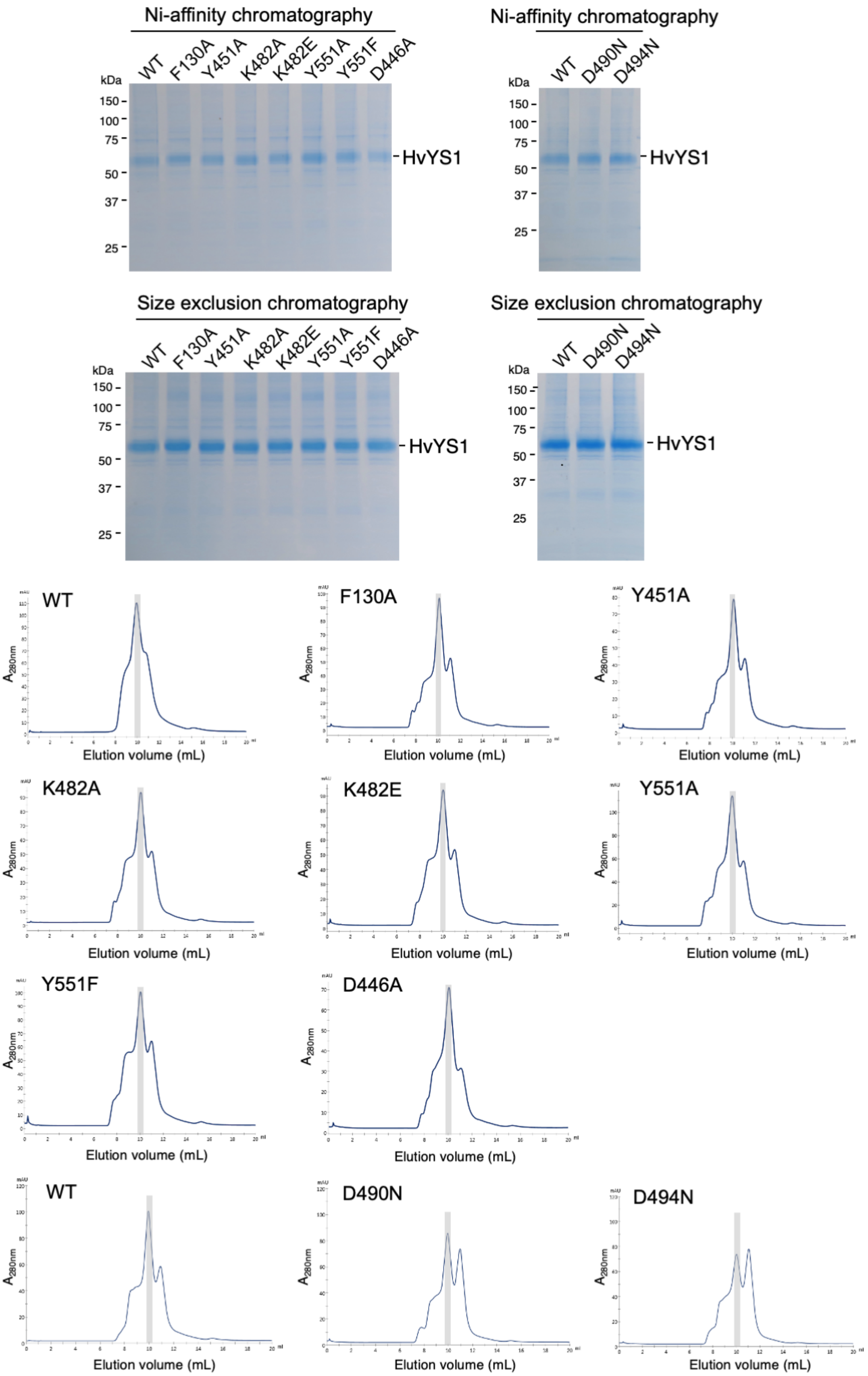

Supplementary Figure 15. Expression and purification of the mutant HvYS1s

SDS-PAGE gels of the wild-type and the mutant HvYS1s after Ni-affinity purification and SEC. SEC profiles of HvYS1s. The peak fractions of the dimeric HvYS1, shown in the shadowed area, were analyzed by SDS-PAGE as described above. Experiments were repeated twice independently with similar results.

Supplementary Figure 1a

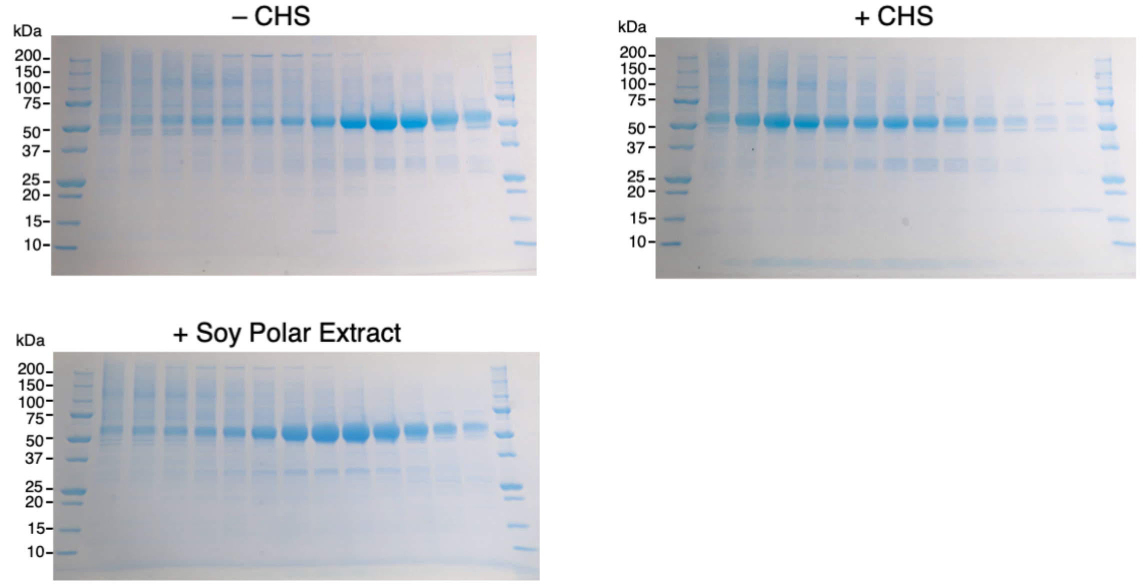

Supplementary Figure 6a

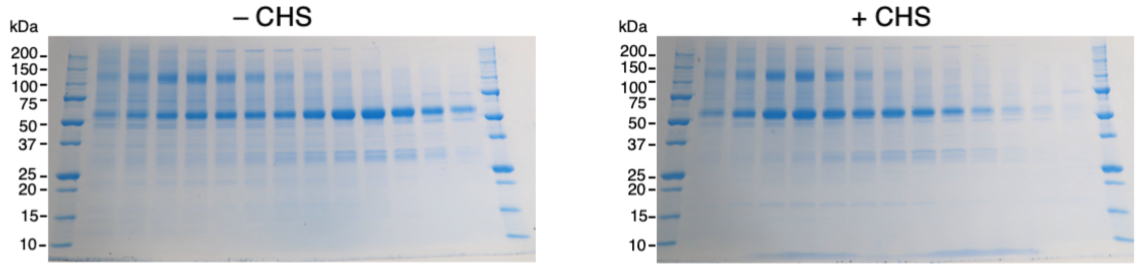

Supplementary Figure 6b

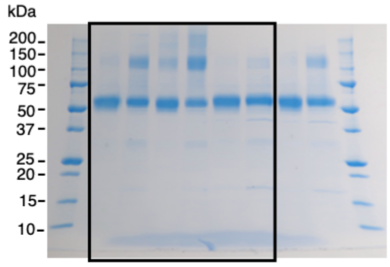

Supplementary Figure15

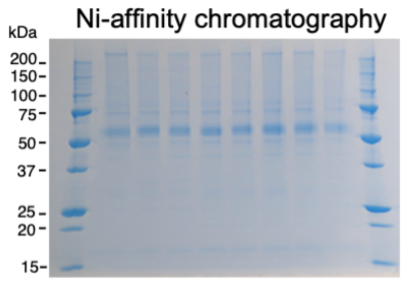

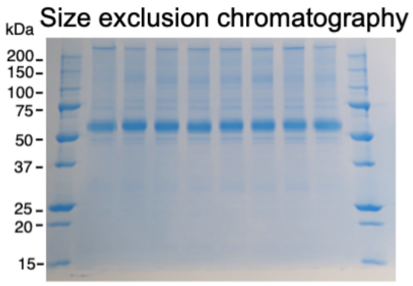

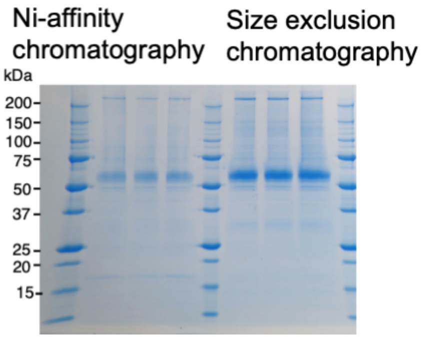

Supplementary Figure 16. Uncropped images of gels

Experiments were repeated twice independently with similar results.

Supplementary Table1. Cryo-EM data

	**Apo**<br><br>**(7WSR)**	**Fe(III)–DMA-bound**<br>**(7WST)**	**Fe(III)–PDMA-bound**<br>**(7WSU)**
Data collection and processing			
Magnification	105,000	105,000	105,000
Voltage (kV)	300	300	300
Total dose (e^-/Å ^{2})	50.3	51.4	49.8
Defocus range (μm)	0.8 - 2.5	0.6 - 2.5	0.8 - 2.5
Pixel size (Å)	0.8285	0.8285	0.8285
Symmetry imposed	*C*2	*C*2	*C*2
Initial particle images (no.)	2,276,022	6,213,206	3,041,199
Final particle images (no.)	319,121	471,603	386,186
Map resolution (Å)	2.9	2.7	2.9
FSC threshold	0.143	0.143	0.143
Map resolution range	2.4 – 3.4	2.2 – 3.2	2.5 – 3.5
Refinement			
Initial model used	None		
Model resolution (Å)			
FSC 0.143, unmasked/masked	2.90/2.87	2.73/2.69	2.92/2.88
Model composition			
Non-hydrogen atoms	9366	9438	9440
Protein residues	1200	1202	1202
Ligands	4	8	8
B factors (Å^2)			
Protein (Å^2)	55.29	45.40	48.82
Ligands (Å^2)	39.38	34.72	38.63
RMS deviation			
Bond length (Å)	0.002	0.003	0.003
Bond angle (°)	0.505	0.612	0.517
Molprobity score	1.32	1.35	1.29
Clash score	5.90	6.39	5.45
Rotamer outliers (%)	0	0	0
Ramachandran plot			
Favored (%)	98.15	98.16	98.32
Allowed (%)	1.85	1.84	1.68
Disallowed (%)	0	0	0

Supplementary Table2. The rmsd of the Fe–ligand distances in MD simulations

System	Chain	Protonation state		Fe-O1	Fe-O4	Fe-O6	Fe-O8	Fe-N1	Fe-N2
Experimental structure			[Å]	2.001	1.968	1.982	2.021	2.513	2.485
1	A	D446p/D490-/	Average [Å]	2.124	2.099	2.105	2.078	2.525	2.553
		D494-/D651-	Std. dev. [Å]	0.049	0.049	0.049	0.049	0.047	0.048
	B	D446p/D490p/	Average [Å]	2.099	2.097	2.085	2.064	2.526	2.552
		D494-/D651-	Std. dev. [Å]	0.049	0.049	0.050	0.050	0.048	0.049
2	A	D446p/D490-/	Average [Å]	2.125	2.102	2.104	2.078	2.526	2.553
		D494p/D651-	Std. dev. [Å]	0.048	0.049	0.049	0.049	0.047	0.048
	B	D446p/D490-/	Average [Å]	2.103	2.087	2.089	2.053	2.515	2.543
		D494-/D651p	Std. dev. [Å]	0.052	0.050	0.053	0.051	0.049	0.051
3	A	D446-/D490-/	Average [Å]	2.121	2.094	2.106	2.069	2.524	2.553
		D494-/D651-	Std. dev. [Å]	0.049	0.049	0.049	0.049	0.047	0.049
	B	D446-/D490p/	Average [Å]	2.124	2.140	2.102	2.067	2.527	2.537
		D494-/D651-	Std. dev. [Å]	0.049	0.048	0.048	0.049	0.048	0.048
4	A	D446-/D490-/	Average [Å]	2.117	2.100	2.106	2.070	2.527	2.555
		D494p/D651-	Std. dev. [Å]	0.048	0.049	0.049	0.048	0.048	0.048
	B	D446-/D490-/	Average [Å]	2.121	2.103	2.109	2.074	2.527	2.553
		D494-/D651p	Std. dev. [Å]	0.049	0.049	0.049	0.049	0.047	0.048
5	A	D446-/D490p/	Average [Å]	2.121	2.132	2.113	2.067	2.531	2.537
		D494p/D651-	Std. dev. [Å]	0.049	0.048	0.049	0.049	0.048	0.048
	B	D446p/D490p/	Average [Å]	2.120	2.101	2.104	2.075	2.524	2.552
		D494p/D651p	Std. dev. [Å]	0.048	0.049	0.049	0.049	0.047	0.048
6	A	D446-/D490p	Average [Å]	2.097	2.098	2.090	2.063	2.526	2.543
		/D494-/D651p	Std. dev. [Å]	0.051	0.050	0.050	0.050	0.048	0.051
	B	D446-/D490-	Average [Å]	2.123	2.099	2.101	2.073	2.525	2.556
		/D494p/D651p	Std. dev. [Å]	0.049	0.049	0.049	0.049	0.047	0.048
all			Average [Å]	2.116	2.104	2.101	2.069	2.525	2.549
			Std. dev. [Å]	0.050	0.051	0.050	0.050	0.048	0.049

References

1. Larkin, M.A. et al. Clustal W and Clustal X version 2.0. *Bioinformatics* **23**, 2947-8 (2007).
2. Robert, X. & Gouet, P. Deciphering key features in protein structures with the new ENDscript server. *Nucleic Acids Res* **42**, W320-4 (2014).
