# Supplementary material for: Uptake mechanism of iron-phytosiderophore from the soil based on the structure of yellow stripe transporter
Source: Nat Commun. 2022 Nov 23;13:7180. doi: 10.1038/s41467-022-34930-1 (PMC9691689; doi:10.1038/s41467-022-34930-1)
Supplement: Supplementary file 2 — Peer Review File [file 41467_2022_34930_MOESM2_ESM.pdf]

nature portfolio

Peer Review File

Uptake mechanism of iron-phytosiderophore from the soil based on the structure of Yellow Stripe transporter

Open Access This file is licensed under a Creative Commons Attribution 4.0 International License, which permits use, sharing, adaptation, distribution and reproduction in any medium or format, as long as you give appropriate credit to the original author(s) and the source, provide a link to the Creative Commons license, and indicate if changes were made. In the cases where the authors are anonymous, such as is the case for the reports of anonymous peer reviewers, author attribution should be to 'Anonymous Referee' followed by a clear attribution to the source work. The images or other third party material in this file are included in the article's Creative Commons license, unless indicated otherwise in a credit line to the material. If material is not included in the article's Creative Commons license and your intended use is not permitted by statutory regulation or exceeds the permitted use, you will need to obtain permission directly from the copyright holder. To view a copy of this license, visit http://creativecommons.org/licenses/by/4.0/.

Reviewers' Comments:

Reviewer #1:
Remarks to the Author:
This manuscript describes the structural characterisation of a mugineic acid (MA), a phytosiderophore, uptake system important for the transport of iron into plants in calcareous soils. These soils have elevated pH and make up a large proportion of potentially arable land. Therefore, understanding the structural basis of MA selectivity and the mechanism of MA uptake could lead to the development of enhanced fertilizers to correct for iron deficiency.

In this manuscript, the apo and substrate-bound states of HvYS1 are captured using cryoEM revealing a new fold (according to the author's DALI search), but with a familiar arrangement seen in other transporters that use an elevator like mechanism. The structures reveal the structural basis of substrate recognition and, while only the outward-facing state was captured, repeat swap modelling suggests an elevator-like mechanism. The structural data are complemented with in vivo functional studies and MD simulations, which combines nicely to reveal mechanistic insight into this interesting transporter.

Overall, this is an intriguing system to study with potential impact that will be of interest to the reader. The structural studies seem well done (although not my field of expertise) and for the most part presented clearly. The functional assays are restricted to testing the effects of mutations to potential binding site residues, so are fairly limited but would sufficiently support the structural work. However, these functional assays require some additional work, detailed below, to support the conclusions currently drawn from them.

Main issues
The functional assays reported in Fig 3f suggests that mutation of any of the residues selected results in no measurable transport activity by HvYS1. The authors suggest that this lack of activity demonstrates that this is a key binding site residue and that mutating these residues prevents substrate binding (or H+ binding for D146A). However, this same result could be obtained if these mutations simply destabilize the protein. Therefore, the authors need to demonstrate that these mutants are properly folded and stable in order for this functional assay to support their conclusions. For example, each mutant could be purified and analyzed by SEC or using the thermal stability assay described the manuscript. Alternatively or in addition, the authors should show that the mutant proteins are expressed and correctly located in the plasma membrane.

Line 134 and Extended data fig 5. The authors performed a multiple sequence alignment and describe some of the hydrophobic residues and the cysteine residue at the dimer interface as highly conserved based on this alignment. However, this alignment is composed of only 4 proteins that look to be >50% identical to each other. Therefore, from the data presented it is not possible to say that any of these residues are particularly well conserved because from the alignment it looks like ~50% of the residues are well conserved. For this type of analysis to be meaningful then the alignment should include a wider selection of sequences.

Other issues
The authors use a thermostability based assay to demonstrate that CHS is able to bind and its presence increases the thermal stability of the protein. However, this stability may be due to some other indirect interactions, e.g. the formation of a detergent/lipid mixed micelle increasing protein stability. The authors could rule out this possibility by performing a control experiment alongside the one presented that includes a different lipid to CHS that would presumably lead to no enhancement of stability.

Line 141 and Extended Figure 6. There is an attempt to confirm the dimer interface location by substituting lysine 673 for a cysteine. However, it is not as convincing as it could be because the vast

majority of the protein in the gel is not crosslinked. Can the authors explain this? The crosslinking reaction was not described in the methods section so I assume that this dimer band is the formed spontaneously without the addition of catalysts. I think how the protein is treated prior to loading on the gel is important to mention in the text for clarity. Perhaps the authors could consider adding a catalyst, e.g. copper phenanthroline or HgCl2, to enhance the formation of crosslinked dimer. In addition, the authors could show crosslinked dimer formation using SEC in the absence of CHS; the crosslinked protein should run as a dimer regardless of the presence or absence of CHS.
Whether the protein is able to form a dimer is really secondary to whether it is a functional dimer. To that end, the authors could determine conditions to fully crosslink the protein and then assess its transport activity under those conditions.

The authors should describe the rationale behind adding the cholesterol hemisuccinate (CHS) to the purification, since it was essential to the formation of the dimeric state. Was its selection part of a wider screen? In addition, the authors mention 4 CHS molecules are bound, but do not detail all of the locations. I think this would be interesting information to include.

Line 108. The authors refer to "an atypical helix hairpin structure". However, there are several examples of helix hairpin structures in other transporters. It is not clear from the text or figures how this hairpin structure is different to those already described in the literature. If it is different then the differences need to be made clear, if it is not then the description as atypical should be removed.

Fig 2. The location of the CHS in the dimer interface is very interesting. However, I think the location could be more clearly demonstrated with a wider angle image of the entire in the dimer. From Fig 1d there looks to be a wide gap between the protomers, but I assume that is filled with only the CHS molecule? I think it would be beneficial to show this, if it is the case.

The authors demonstrate that the oligomeric state of the protein can be controlled by the presence of CHS. Almost all other elevator-like transporters described form oligomers. Could the authors comment on the possibility of this protein working as a monomer? Does the concentration of the CHS used in the purification reflect the concentration of CHS or related lipid in the natural membrane?

I could not find a description of what the control assay was in the transport assays shown in Fig. 3f, which is important information to include.

Reviewer #2:
Remarks to the Author:
The manuscript by Yamagata et al. presents three cryo-EM structures of YS1 transporter, an important iron reabsorption system in grasses with hundreds of paralogous proteins in different plant species that so far lack structural characterisation. Of note, YS1 systems are the target of potent fertilisers to aid global production of major crops like rice and maize.
The work by Yamagata et al. represents the first structural characterisation of a YS1 transport system and will be of great interest to the membrane transport, and plant physiology communities. Notably, using structural arguments, Yamagata et al. propose a very clear and well-justified transport mechanism, whereby large elevator-like movements of the transport domain translocate Fe(III)-DMA substrate across the membrane, using the transmembrane proton gradient as energy source.
The cryo-EM maps and structures are of very-high quality, the text is concise and clear, and the figures nicely done to support the scientific claims. I congratulate the authors for a thorough and important piece of work.

I have minor comments that aim to improve the manuscript clarity:

Tittle. The manuscript shows experimentally-determined structures of HvYS1 in apo and substrate-

bound outward facing states. It also predicts conformational changes to isomerize HvYS1 into the inward-facing state to translocate the substrate. Finally, it highlights the role of D446 to thermodynamically couple protons to substrate transport. All this constitutes a clear and well-supported-by-the-data structural transport mechanism of these important group of proteins. Please, consider changing the tittle accordingly to something like: "Uptake mechanism of iron-phytosiderophore from the soil"

Line 58: " Nicotinamine ……. forms the major structure of MAS" what is the meaning of "major structure" in this context?

Line 102: "one is the alpha-helical core domain called YS core domain, while the other one is a scaffold domain…" both core and scaffold domains are mostly alpha-helical, with the exception of interfacial S1. Please, consider removing "alpha-helical" from the sentence.

Line 115: "The cross brace of "M" and "W" shaped repeats suggest the structural rigidity of the scaffold domains". Please, explain more clearly what "cross brace" means in this context and why it suggests structural rigidity. Would a structural comparison between core-scaffold versus scaffold-scaffold interfaces suggests that the former would enable core-domain movements, while the latter would preclude scaffold domain movements (rigidity)?

CHS molecules stabilize HvYS1 dimer. This is a very interesting result. It would be nice to see the position and orientation of the modelled CHS molecules in the context of the overall dimeric structure (that is in Fig. 1d). Line 139: "supporting cholesterol-mediated dimer of HvSY1 in the native membrane environment". Is cholesterol the most abundant lipid-sterol in barley cells, so that the above-mentioned statement is justified? Finally, the stabilizing role of lipids at oligomeric interfaces of membrane proteins has been studied in some detail in the literature (e.g. Gupta et al., Nature 2017), and it would be interesting to put HvYS1 results in the context of previous findings, or at least cite previous work.

Extended Data Fig. 6. The "cross-linking" experiment in this figure is rather inconclusive. From panel a in that figure, it is clear that in solution both WT and K673C are mainly in dimeric form. The cross-linked band observed by SDS-PAGE is a relatively minor part of the total protein, and it is not fully reverted by application of DTT. Would it be possible that the crosslinked product, at least partly, is due to addition of a denaturing agent (SDS) to the sample? I couldn't find the details of these experiments in the method section, but do SDS samples contain high-concentrations of cysteine-alkylating agents (like NEM) to prevent SDS-induced crosslinking? One way to avoid experiments containing SDS, and assay crosslinking of folded proteins, could be to compare the SEC profiles in non-denaturing LMNG-based buffers of WT and K673C mutant in the absence of CHS, as in Extended Data Fig. 1a.

DMA binding. Line 154: "Fe(III)-DMA density is found at the extracellular gate in the YS core domain.." Please clarify what is considered the extracellular gate and why? Is it H7? Is there evidence that the so-called gate is dynamic and gates the DMA-binding pocket? Moreover, from a mechanistic point of view, it is likely important that the transporter selectively binds Fe(III)-DMA over apo-DMA to avoid futile cycles and proton spill. Does the structure shed any light on why HvYS1 would not bind apo-DMA? The discussion section in the manuscript is rather minimal, and it would be interesting to include insights on important aspects of the transport mechanism, like Fe(III)-DMA vs apo-DMA binding.

Ext Data Figures with cryo-EM analysis pipelines. Consider adding scale bars to all these figures in the representative micrographs.

Line 204 onward. Please, consider changing the tittle of this section from "The proposed elevator-like transport mechanism" to "Elevator-like transport mechanism". More importantly, it is unclear why the authors discarded roles of D270 and D617 in proton coupling, on the basis that they "formed a

hydrogen bond and thereby are assumed to be protonated". The other four aspartate-residue candidates also appear to be involved in H-bonding and yet, they were considered as proton-binding candidates. Moreover, the Fe(III)-DMA bound YS1 structure likely represents a protonated state of the cycle, so the proton-acceptor candidate(s) is(are) expected to be protonated in such state. Also around line 211, in the text there is no structural explanation on why D446 protonation is essential for Fe(III)-DMA binding.

Maybe, this part of the manuscript would be clearer if the authors present first the elevator-like mechanism with an associated structural figure (current Fig. 4c), and then they present the proton-coupled mechanism with a separate associated figure in the main text including the position of the 6 aspartate-residue candidates and MD simulations, as well as more detail explanations in the text on why D270 and D617 should not be considered as proton acceptors, and why protonated D446 stabilises Fe(III)-DMA binding. The cartoon in Fig. 4b is very helpful and elegant and I would consider keeping it as the final figure of the manuscript (revised Fig. 6). Please, consider the above-mentioned rearrangement as a suggestion. I do leave the final decision on figure arrangements to the authors.

Re: NCOMMS-22-22936-T

Point-by-point responses

We are grateful to the reviewers for their helpful comments and made every effort to improve the manuscript according to their comments. We hope that our edits and the responses we provide below satisfactorily address all the issues and concerns the reviewers have noted.

Reviewer #1:

1. Main issues

   The functional assays reported in Fig 3f suggests that mutation of any of the residues selected results in no measurable transport activity by HvYS1. The authors suggest that this lack of activity demonstrates that this is a key binding site residue and that mutating these residues prevents substrate binding (or H+ binding for D146A). However, this same result could be obtained if these mutations simply destabilize the protein. Therefore, the authors need to demonstrate that these mutants are properly folded and stable in order for this functional assay to support their conclusions. For example, each mutant could be purified and analyzed by SEC or using the thermal stability assay described the manuscript. Alternatively or in addition, the authors should show that the mutant proteins are expressed and correctly located in the plasma membrane.

   According to the suggestions, we first confirmed the similar surface expression level of the wild-type and the mutant HvYS1s on the insect cell membrane using immunofluorescence (Supplementary Fig. 13). In addition, we analyzed the expression level and the proper folding

of the mutant HvYS1s by Ni-affinity purification and the subsequent SEC analysis from the same volume of the Sf9 cultured cells (Supplementary Fig. 14).

2. Line 134 and Supplementary fig 5. The authors performed a multiple sequence alignment and describe some of the hydrophobic residues and the cysteine residue at the dimer interface as highly conserved based on this alignment. However, this alignment is composed of only 4 proteins that look to be >50% identical to each other. Therefore, from the data presented it is not possible to say that any of these residues are particularly well conserved because from the alignment it looks like ~50% of the residues are well conserved. For this type of analysis to be meaningful then the alignment should include a wider selection of sequences.

   We newly added three more sequences of the YS/YSL family proteins into the multiple sequence alignment (revised Supplementary Fig. 5). The identical residues in the new alignment are 273 residues (~40% of HvYS1), whereas those in the previous alignment are 354 residues (~52% of HvYS1). Three newly added YS/YSL proteins are NA-specific transporters. Therefore, the residues involved in the substrate binding are not perfectly conserved in the new alignment. We have provided a more detailed description of the sequence conservation on the substrate-binding residues on Page 9, Line 187.

3. Other issues

   The authors use a thermostability based assay to demonstrate that CHS is able to bind and its presence increases the thermal stability of the protein. However, this stability may be due to some other indirect interactions, e.g. the formation of a detergent/lipid mixed micelle increasing protein stability. The authors could rule out this possibility by performing a

control experiment alongside the one presented that includes a different lipid to CHS that would presumably lead to no enhancement of stability.

We thank the reviewer for the great suggestion. We tested soy extract polar lipid as a different lipid to CHS. As expected, HvYS1 purified in the presence of soy extract polar lipid did not form a dimer in SEC and showed no enhancement of thermal stability (revised Supplementary Fig. 1). We have described it on Page 5, Line 91.

4. Line 141 and Extended Figure 6. There is an attempt to confirm the dimer interface location by substituting lysine 673 for a cysteine. However, it is not as convincing as it could be because the vast majority of the protein in the gel is not crosslinked. Can the authors explain this? The crosslinking reaction was not described in the methods section so I assume that this dimer band is the formed spontaneously without the addition of catalysts. I think how the protein is treated prior to loading on the gel is important to mention in the text for clarity. Perhaps the authors could consider adding a catalyst, e.g. copper phenanthroline or HgCl2, to enhance the formation of crosslinked dimer. In addition, the authors could show crosslinked dimer formation using SEC in the absence of CHS; the crosslinked protein should run as a dimer regardless of the presence or absence of CHS. Whether the protein is able to form a dimer is really secondary to whether it is a functional dimer. To that end, the authors could determine conditions to fully crosslink the protein and then assess its transport activity under those conditions.

We apologize that the description of the experimental procedures on our crosslinking reaction was not satisfactory. First, as the reviewer assumed, the crosslink of HvYS1^{K673C}

was spontaneously formed, and thereby most of the protein in the gels was not crosslinked. According to the suggestions, we confirmed an enhancement of crosslink by adding a catalyst, $CuCl_2$ (revised Supplementary Fig. 6b). In addition, we analyzed HvYS1^{K673C} in the absence of CHS, and verified the crosslinked dimer formation, as the reviewer expected (revised Supplementary Fig. 6a). We have described it on Page 7, line 144. In addition, we have provided a full description of the method of our disulfide crosslinking experiment (Page 13, Line 334).

We agree on the importance to test the transport activity of HvYS1^{K673C} under the condition of fully crosslinking the protein. Although $CuCl_2$ could enhance the crosslinked dimer in our experiment, copper ions can be chelated by DMA as well as Fe^{3+} and therefore our insect cell assay system cannot be performed with the excess of $CuCl_2$.

5. The authors should describe the rationale behind adding the cholesterol hemisuccinate (CHS) to the purification, since it was essential to the formation of the dimeric state. Was its selection part of a wider screen? In addition, the authors mention 4 CHS molecules are bound, but do not detail all of the locations. I think this would be interesting information to include.

We added CHS in the purification step because cholesterol was well known to modulate the stability and function of membrane proteins including GPCRs and ion channels {Duncun et al. 2020, Annu. Rev. Pharmacol. Toxicol.}. Therefore, the positive effect of CHS on HvYS1 was not found from a wider screening. We have described it on Page 15, Line 306.

According to the suggestion, we have shown the residues interacting with the CHS at the dimer interface in the revised Fig 2c. We have also provided a detailed view of the interaction between HvYS1 and the second CHS in Supplementary Fig. 1c.

6. Line 108. The authors refer to “an atypical helix hairpin structure”. However, there are several examples of helix hairpin structures in other transporters. It is not clear from the text or figures how this hairpin structure is different to those already described in the literature. If it is different then the differences need to be made clear, if it is not then the description as atypical should be removed.

   We agree that the description of “an atypical helix hairpin structure” was a bit confusing. We have provided a more detailed description on Page 6, Line 111, and structural comparison with the helical hairpins in the other elevator-like transporters (Supplementary Fig. 4b).

7. Fig 2. The location of the CHS in the dimer interface is very interesting. However, I think the location could be more clearly demonstrated with a wider angle image of the entire in the dimer. From Fig 1d there looks to be a wide gap between the protomers, but I assume that is filled with only the CHS molecule? I think it would be beneficial to show this, if it is the case.

   According to the suggestion, we have shown the modeled CHS in the entire HvYS1 dimer (revised Fig 1d). The wide gap beneath the dimer interface is occupied by the lipid-like densities in the cryo-EM map (revised Supplementary Fig. 1d), although we could not model the lipid molecules to those densities due to the ambiguity.

8. The authors demonstrate that the oligomeric state of the protein can be controlled by the presence of CHS. Almost all other elevator-like transporters described form oligomers. Could the authors comment on the possibility of this protein working as a monomer? Does the concentration of the CHS used in the purification reflect the concentration of CHS or related lipid in the natural membrane?

We really appreciate the comments. HvYS1 is likely to be functional as a dimer, because CHS remarkably enhances the thermal stability of HvYS1 in our experiment. However, HvYS1 should be in the equilibrium between dimer and monomer in the membrane in our insect cell-based assay system. In an elevator-like transport model, the transport activity is not necessary to be coupled with the oligomerization, though the oligomerization might contribute to the structural rigidity as a scaffold. Taken together, we could not rule out the possibility of HvYS1 working as a monomer in our experiment. We have described it on Page 13, Line 266. The concentration of the CHS used in our experiment was dependent on the solubility of CHS and not related to the concentration in the natural membrane.

9. I could not find a description of what the control assay was in the transport assays shown in Fig. 3f, which is important information to include.

Thanks for pointing this out. We have provided the information about the control in our transport assay in the figure legend of Fig. 3F (Page 36, Line 772).

Reviewer #2 (Remarks to the Author):

1. Tittle. The manuscript shows experimentally-determined structures of HvYS1 in apo and substrate-bound outward facing states. It also predicts conformational changes to isomerize HvYS1 into the inward-facing state to translocate the substrate. Finally, it highlights the role of D446 to thermodynamically couple protons to substrate transport. All this constitutes a clear and well-supported-by-the-data structural transport mechanism of these important group of proteins. Please, consider changing the tittle accordingly to something like: "Uptake mechanism of iron-phytosiderophore from the soil"

   We appreciate the reviewer's positive comments. We changed the title as follows: "Uptake mechanism of iron-phytosiderophore from the soil based on the structure of Yellow Stripe transporter"

2. Line 58: " Nicotinamine ……. forms the major structure of MAS" what is the meaning of "major structure" in this context?

   Thanks for pointing this out. This is a simple mistake that we forgot to erase the sentence "and forms a major structure". We have removed it (Page 3, Line 57).

3. Line 102: "one is the alpha-helical core domain called YS core domain, while the other one is a scaffold domain…" both core and scaffold domains are mostly alpha-helical, with the exception of interfacial S1. Please, consider removing "alpha-helical" from the sentence.

   Thanks for the comment. We agree and have removed "alpha-helical" from the sentence as suggested (Page 5, Line105).

4. Line 115: “The cross brace of “M” and “W” shaped repeats suggest the structural rigidity of the scaffold domains”. Please, explain more clearly what “cross brace” means in this context and why it suggests structural rigidity. Would a structural comparison between core-scaffold versus scaffold-scaffold interfaces suggests that the former would enable core-domain movements, while the latter would preclude scaffold domain movements (rigidity)?

We agree that the original sentence was a bit confusing and have revised it as follows; “The “M”- and “W”-shaped repeats cross each other to form extensive contact between them.”(Page 6, Line 120). We have also revised Supplementary Fig. 4c to show the interactions between two repeats.

5. CHS molecules stabilize HvYS1 dimer. This is a very interesting result. It would be nice to see the position and orientation of the modelled CHS molecules in the context of the overall dimeric structure (that is in Fig. 1d). Line 139: “supporting cholesterol-mediated dimer of HvSY1 in the native membrane environment”. Is cholesterol the most abundant lipid-sterol in barley cells, so that the above-mentioned statement is justified? Finally, the stabilizing role of lipids at oligomeric interfaces of membrane proteins has been studied in some detail in the literature (e.g. Gupta et al., Nature 2017), and it would be interesting to put HvYS1 results in the context of previous findings, or at least cite previous work.

We really appreciate the great suggestions. We showed the modeled CHS in the entire HvYS1 dimer in the revised Fig. 1d. Intriguingly, the plant has a wide variety of plant-specific sterols (phytosterols). In barley, phytosterols are highly enriched in the plasma membrane

{Rochester, et al. 1987 Arch. Biochem. Biophys.; Brown, et al. 1989 Plant Physiol.}. We have discussed it on Page 13, Line 259.

In the context of the previous work by Gupta et al., HvYS1 has a weak dimer interface based on the buried surface area. The essential role of CHS in stabilizing HvYS1 dimer is consistent with the hypothesis by Gupta et al. that the interfacial lipids are critical for stabilizing the weak dimer. We have discussed it on Page 13, Line 263.

6. Supplementary Fig. 6. The "cross-linking" experiment in this figure is rather inconclusive. From panel a in that figure, it is clear that in solution both WT and K673C are mainly in dimeric form. The cross-linked band observed by SDS-PAGE is a relatively minor part of the total protein, and it is not fully reverted by application of DTT. Would it be possible that the crosslinked product, at least partly, is due to addition of a denaturing agent (SDS) to the sample? I couldn't find the details of these experiments in the method section, but do SDS samples contain high-concentrations of cysteine-alkylating agents (like NEM) to prevent SDS-induced crosslinking? One way to avoid experiments containing SDS, and assay crosslinking of folded proteins, could be to compare the SEC profiles in non-denaturing LMNG-based buffers of WT and K673C mutant in the absence of CHS, as in Supplementary Fig. 1a.

We apologize that the description of our crosslinking experiment and the original supplementary Fig. 6 were a bit confusing. The crosslinked dimer of HvYS1^{K673C} in the original figure was spontaneously formed and therefore most of the proteins were not crosslinked. We have revised the text on Page 7, Line 144.

We confirmed that the crosslinked dimer was enhanced by the addition of $CuCl_2$ (revised Supplementary Fig. 6b, Page 7, Line 146). Moreover, the crosslinked dimer was almost completely reversed by the addition of 50 mM DTT, a 10 times higher concentration than the original experiment (revised Supplementary Fig. 6b, Page 7, Line 147). Although we did not add the cysteine-alkylating reagent to prevent SDS-induced crosslinking, the observed crosslinking was likely due to the dimer formation but not denaturation, as the wild-type protein was not crosslinked. We have provided a full description of the method of our disulfide crosslinking experiment (Page 13, Line 334).

According to the suggestion, we analyzed SEC of HvYS1^{K673C}without CHS (revised Supplementary Fig 6a). The crosslinked dimer by K673C mutation is maintained even in the absence of CHS (Page 7, Line 148).

7. DMA binding. Line 154: "Fe(III)-DMA density is found at the extracellular gate in the YS core domain.." Please clarify what is considered the extracellular gate and why? Is it H7? Is there evidence that the so-called gate is dynamic and gates the DMA-binding pocket? Moreover, from a mechanistic point of view, it is likely important that the transporter selectively binds Fe(III)-DMA over apo-DMA to avoid futile cycles and proton spill. Does the structure shed any light on why HvYS1 would not bind apo-DMA? The discussion section in the manuscript is rather minimal, and it would be interesting to include insights on important aspects of the transport mechanism, like Fe(III)-DMA vs apo-DMA binding.

Thanks for the reviewer's comments. We agree the word "gate" is misleading. We have removed it (Page 8, Line 160). We have discussed the possibility of binding to apo-DMA on

Page 13, Line 273. In brief, we consider it is unlikely as the apo-DMA should form the elongated conformation and cannot maintain the specific interaction with HvYS1.

8. Ext Data Figures with cryo-EM analysis pipelines. Consider adding scale bars to all these figures in the representative micrographs.

   Thanks for pointing this out. We have added scale bars in the revised Supplementary Fig. 2, Supplementary Fig. 7, and Supplementary Fig. 10.

9. Line 204 onward. Please, consider changing the tittle of this section from "The proposed elevator-like transport mechanism" to "Elevator-like transport mechanism". More importantly, it is unclear why the authors discarded roles of D270 and D617 in proton coupling, on the basis that they "formed a hydrogen bond and thereby are assumed to be protonated". The other four aspartate-residue candidates also appear to be involved in H-bonding and yet, they were considered as proton-binding candidates. Moreover, the Fe(III)-DMA bound YS1 structure likely represents a protonated state of the cycle, so the proton-acceptor candidate(s) is(are) expected to be protonated in such state. Also around line 211, in the text there is no structural explanation on why D446 protonation is essential for Fe(III)-DMA binding.

   Maybe, this part of the manuscript would be clearer if the authors present first the elevator-like mechanism with an associated structural figure (current Fig. 4c), and then they present the proton-coupled mechanism with a separate associated figure in the main text including the position of the 6 aspartate-residue candidates and MD simulations, as well as more detail explanations in the text on why D270 and D617 should not be considered as proton acceptors,

and why protonated D446 stabilises Fe(III)-DMA binding. The cartoon in Fig. 4b is very helpful and elegant and I would consider keeping it as the final figure of the manuscript (revised Fig. 6). Please, consider the above-mentioned rearrangement as a suggestion. I do leave the final decision on figure arrangements to the authors.

According to the suggestion, we have changed the title to "Elevator-like transport mechanism". Then, we divided the original paragraph into "Elevator-like transport mechanism" and "Proton-coupled transport". Accordingly, we have moved the original Fig. 4c to Figure 4. We have moved the original Supplementary Fig. 12a, the original Figure 4a, and 4b, to the revised Figure 5a, 5b, and 5c, respectively.

We agree that the reason why we excluded Asp270 and Asp617 is a bit confusing. Asp270–Asp617 pair form a hydrogen bond in which one of them should be protonated. By contrast, the other acidic residues are involved in hydrogen bonding with the amine or the hydroxyl group and thereby are assumed to be deprotonated. More importantly, Asp270 and Asp617 are in the scaffold domain, unlikely to be involved in proton-binding based on the key features of elevator transporters (Fig. 1f). Taken together, we excluded these two Asp residues from a potential proton-binding site. We have provided a more detailed description on Page 11, Line 233.

Although we intended to explain why the protonation at Asp446 stabilizes Fe–DMA-bound state from the cryo-EM structure and MD simulations, we could not find a clear answer from the simple H-bonding network around Asp446. One of the possibilities is that the negative charge of the deprotonated Asp446 may slightly destabilize the substrate binding site in the C-terminal region of H6, which has a negative dipole charge (Supplementary Fig. 12d). We have described it on Page 14, Line 278.

Reviewers' Comments:

Reviewer #1:
Remarks to the Author:
The authors have done an excellent job with this revision of their manuscript and have addressed all of the issues raised in my initial review.
All of the additional experiments are clearly described and are very well executed and presented.

Reviewer #2:
Remarks to the Author:
The authors have made an honest and successful effort to improve the manuscript, and I don't have any additional comments, beyond some typos (see below). Once again, congratulations on a very nice piece of work.

Line 101: "...are present between dimers...." It should probably read "between protomers" or "at the dimeric interface"

Line 102: "The beneath of the two subunits..." should probably read : "The cytoplasmic part of the two subunits"

Reviewer #3:
Remarks to the Author:
Yamagata and coworkers present cryo-EM structures of the yellow stripe transporter, in combination with different experimental assays, to propose a mechanism for binding of the iron-phytosiderophore, proton coupling and the elevator-like conformational changes associated to transport. In addition, they perform molecular dynamics simulations to give further insights into the proton-coupling mechanism.

Although the simulations are a very nice complement of the static cryo-EM structures, there are some issues that need to be addressed before the manuscript can be considered for publication.

(1) It is unclear why the authors considered only the six Asp residues mentioned in the manuscript as possible proton binding sites. Aren't there other titratable residues (Glu/His) within the YS core domain potentially involved in proton coupling (either as proton binding sites or as a waystation for proton transfer)? is there any experimental evidence (e.g. mutagenesis) supporting the choice of these Asp residues?

(2) In lines 465-467 it is described that the membrane was composed of only POPC. Why were the cholesterol molecules involved in dimer formation not included?

(3) The authors should double-check the name/version of the CHARMM parameters used, as well as the references cited in lines 479-481. Reference 64 seems to refer to the protein (CMAP corrections), if I'm not mistaken.

(4) The authors should describe in more detail the coordination sphere of Fe(III). Fe(III) is expected to have octahedral coordination, and from Figure 3e it is difficult to tell if the groups acting as ligands of Fe(III) are all belonging to the DMA phytosiderophore or there is one axial position that could be occupied by a water molecule. Moreover, the authors should show (in the SI) that the Fe-ligand distances are maintained during the simulation (and whether a water molecule can bind to Fe(III), in case the phytosiderophore only provides 5 coordinating groups).

(5) It would be great if the authors could present more details of the simulations with protonated Asp490, Asp494 or Asp651 in the SI. From what I can see in Supplementary Figure S12a, these Asp residues, as well as Asp446, are involved in interhelical H-bonds with Ser/Thr or Asn/Gln and thus their protonation could alter the associated H-bond network and contribute to the conformational changes necessary for transport, as shown for other membrane proteins; see e.g. Bondar, Frontiers in Chemistry (2022), https://doi.org/10.3389/fchem.2021.685761.

Re: NCOMMS-22-10302-A

Point-by-point responses

We are grateful to the reviewers for their helpful comments. As described in the cover letter, we recalculate MD simulations with harmonic restraint to all the Fe–ligand distances, according to the comments from reviewer #3 (please see the point-by-point response to reviewer #3). Our new MD simulations implicated that the protonation at Asp446, Asp490, and Asp494 would contribute to the conformational change necessary for the transport, which was also supported by our mutational analysis. We hope that our edits and the responses we provide below satisfactorily address all the issues and concerns the reviewers have noted.

Reviewer #1:

The authors have done an excellent job with this revision of their manuscript and have addressed all of the issues raised in my initial review.

All of the additional experiments are clearly described and are very well executed and presented.

We really appreciate the reviewer's positive comment.

Reviewer #2:

The authors have made an honest and successful effort to improve the manuscript, and I don't have any additional comments, beyond some typos (see below). Once again, congratulations on a very nice piece of work.

Line 101: "...are present between dimers...." It should probably read "between protomers" or "at the dimeric interface"

Line 102: "The beneath of the two subunits..." should probably read : "The cytoplasmic part of the two subunits"

Thanks for pointing these out. We revised the text according to the reviewer's comments (Page 5, Line101, and 102). We thank the reviewer again for the positive comments.

Reviewer #3:

1. It is unclear why the authors considered only the six Asp residues mentioned in the manuscript as possible proton binding sites. Aren't there other titratable residues (Glu/His) within the YS core domain potentially involved in proton coupling (either as proton binding sites or as a waystation for proton transfer)? is there any experimental evidence (e.g. mutagenesis) supporting the choice of these Asp residues?

   Thanks for pointing this out. We have shown all Asp, Glu, and His residues in the revised Supplementary Fig 12a. Among them, six Asp residues (Asp270, Asp446, Asp490, Asp494, Asp617, Asp651) are on the surface of the central cavity, and three of them (Asp446, Asp490, and Asp494) are in the YS core, likely to be the potential protonation site.

   To experimentally support our MD simulation results, we performed mutational analyses for the D446A, D490N, and D494N mutant proteins, as described in the revised Figure 5f.

2. In lines 465-467 it is described that the membrane was composed of only POPC. Why were the cholesterol molecules involved in dimer formation not included?

   We thank the reviewer for pointing out this important point. We forgot to describe that we relaced CHS with cholesterol in our MD simulations. We have described it on Page 11, Line 244.

3. The authors should double-check the name/version of the CHARMM parameters used, as well as the references cited in lines 479-481. Reference 64 seems to refer to the protein (CMAP corrections), if I'm not mistaken.

   We appreciate the reviewer for pointing this out. We removed reference 64.

4. The authors should describe in more detail the coordination sphere of Fe(III). Fe(III) is expected to have octahedral coordination, and from Figure 3e it is difficult to tell if the groups acting as ligands of Fe(III) are all belonging to the DMA phytosiderophore or there is one

axial position that could be occupied by a water molecule. Moreover, the authors should show (in the SI) that the Fe-ligand distances are maintained during the simulation (and whether a water molecule can bind to Fe(III), in case the phytosiderophore only provides 5 coordinating groups).

In our cryo-EM structure, Fe(III) is octahedrally coordinated by four oxygen atoms (O1, O4, O6, and O8) and two nitrogen atoms (N1 and N2) of DMA (no water molecule is necessary to complete the coordination), consistent with the previous crystal structures of Cu–MA and Co–MA. We have shown the enlarged view of the coordination sphere in the Fe(III)–DMA and the Fe(III)–PDMA from this study, as well as the previously reported Cu(II)–MA and Co(II)–MA structures in the revised Supplementary Fig. 9a.

*The Fe–O distances of Fe–DMA in our cryo-EM structure are about 2.0 Å, whereas the Fe–N distances are about 2.5 Å, longer than that expected for a coordination bond with a nitrogen atom based on the bond-valence sum rule (2.1 Å) (Zheng et al. 2017, Acta Acta Cryst. D**73**, 316–325). Therefore, we imposed harmonic restraints only on the distances between the* Fe^{3+} *ion and the oxygen atoms, with the equilibrium distance of 0.2 nm and a force constant of* 1.0×10^5 *kJ* mol^{-1} nm^{-2} *during simulations. The Fe–O distances were maintained at about 2.1 Å, whereas the Fe–N distances were elongated by 1–1.5 Å. As a result, the Fe(III)–DMA structure was distorted during the MD simulations, leading to a displacement of Fe–DMA from HvYS1.*

We, therefore, redid MD simulations with harmonic restraints on all the Fe–ligand distances. The Fe–ligand distances and the overall structure of Fe–DMA were appropriately maintained during new MD simulations (Supplementary Table 2). The MD simulations with each of Asp446, Asp490, Asp494 protonated resulted in that the Asp494 protonated state is remarkably stable, as the rmsd of the bound Fe(III)–DMA shows minimum fluctuation (Fig. 5c; Supplementary Fig. 12c), suggesting that the Asp494 protonated state structure corresponds to the cryo-EM structure. We further tested the additional protonated states (Asp446/Asp494 protonated, Asp490/Asp494 pronated, and Asp446/Asp490/Asp494 protonated states). The simulation with Asp446/Asp490/Asp494 protonated displays a substantial movement of TM6 (Fig 5c; Supplementary Fig. 12c, Supplementary Fig. 13), which would contribute to the conformational change necessary for the transport. To support

it, the D446A, D490N, and D494N mutant HvYS1 impaired the transport activity, as described in the revised Figure 5f.

5. It would be great if the authors could present more details of the simulations with protonated Asp490, Asp494 or Asp651 in the SI. From what I can see in Supplementary Figure S12a, these Asp residues, as well as Asp446, are involved in interhelical H-bonds with Ser/Thr or Asn/Gln and thus their protonation could alter the associated H-bond network and contribute to the conformational changes necessary for transport, as shown for other membrane proteins; see e.g. Bondar, Frontiers in Chemistry (2022), https://doi.org/10.3389/fchem.2021.685761.

   According to the reviewer's suggestion, we described the movement of the transmembrane helices involved in the H-bond with three Asp residues (Asp446, Asp490, and Asp494) in the revised Supplementary Fig. 13. We also analyzed the conformational changes of the helices in the MD simulations by principal component analysis, described in the revised Supplementary Fig. 12c. As described above, the simulation with Asp446/Asp490/Asp494 protonated displays a TM6 displacement from YS core domain, potentially contributing to the conformational change necessary for the transport.

Reviewers' Comments:

Reviewer #3:
Remarks to the Author:
I thank the authors have considered most of my comments on the molecular dynamics simulations.
The quality of the simulations and their analysis is very much improved and the rationale behind the choice of Asp residues to be protonated is much clearer and further supported by mutagenesis experiments.
Therefore, I believe the manuscript is in good shape for publication.
